# Impedance Inhomogeneity in SiO/Gr Composite Anode

**DOI:** 10.1002/smsc.202300291

**Published:** 2024-06-14

**Authors:** Xiang Gao, Jun Xu

**Affiliations:** ^1^ Department of Mechanical Engineering University of Delaware Newark DE 19716 USA; ^2^ Vehicle Energy & Safety Laboratory (VESL) University of Delaware Newark DE 19716 USA

**Keywords:** finite‐element multiphysics modeling, lithium‐ion batteries, polarization evolution, SiO/Gr composite anode

## Abstract

Silicon/carbon (Si/C) composite anode materials have emerged as promising candidates for high‐energy‐density lithium‐ion batteries (LIBs), boasting advantages such as high capacity, cost‐effectiveness, and abundance. However, the integration of Si‐based materials into conventional graphite anodes introduces heterogeneous interactions between electrochemical and mechanical behaviors, owing to substantial volume changes and chemical potential variations. One significant consequence of these interactions is the impedance inhomogeneity, which adversely affects the discharging capacity of Si‐based LIBs. In an effort to comprehensively understand this phenomenon and its underlying mechanisms, an electrochemo‐mechanical‐coupled model is established, incorporating detailed particle geometries on the anode side. The model is employed to investigate polarization components and their evolution during the charging/discharging process. Various influencing factors, such as SiO weight percentage (wt%), electrode thickness, and SiO distributions (both in terms of distribution uniformity and direction), are systematically discussed. In this study, an efficient computational approach is offered to analyze battery polarizations, deepening the understanding of the inhomogeneous evolution of these polarizations in Si/C composite anodes. Ultimately, these insights guide the design of anodes for next‐generation high‐energy‐density LIBs.

## Graphical abstract

Silicon/carbon anode shows impedance inhomogeneity. An electrochemo‐mechanical‐coupled model is established, incorporating detailed particle geometries of anode. The model is employed to investigate polarization components and their evolution during the charging/discharging process. Various influencing factors, such as SiO weight percentage (wt%), are discussed. In this study, an efficient computational approach is offered to analyze battery polarizations, providing guidance of anode design.

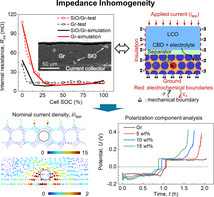

## 1 Introduction

High‐capacity anode materials, such as Si‐based materials^[^ ^1^ ^]^ and Li metal,^[^ ^2^ ^]^ have attracted increasing attention from both academia and industry due to the growing demand for high‐energy‐density solutions, a need that has intensified with the rapid development of lithium‐ion batteries (LIBs) in recent years. Among these emerging anode candidates, Si‐based materials, including Si nanomaterials,^[^ ^3^ ^]^ Si oxides,^[^ ^4^ ^]^ and Si/C composites,^[^ ^5^ ^]^ distinguish themselves as highly promising on a commercial scale due to their cost‐effectiveness,^[^ ^6^ ^]^ earth abundance,^[^ ^7^ ^]^ and substantial theoretical galvanic capacity (up to 4200 and 3860 mAh g^−1^ theoretical capacities for pure Si and Li metal, respectively).^[^ ^8^ ^]^ However, the well‐known challenge stemming from the significant volume change during the electrochemical cycling of Si‐based materials has propelled the investigation and development of Si/C composites into the forefront.^[^ ^9^ ^]^ In these composites, carbon materials (such as hard carbon, graphene, and graphite) play a dual role, serving as both energy‐storage elements and mechanical buffers to address the volume expansion issues. At the current stage, the Si weight percentage (wt%) in the Si/C composite anode is controlled at a low level (≈5–10%) to align with market promotion and facilitate large‐scale fabrication.[^1^, ^10^] Hence, the presence of Si materials resembles numerous “islands” scattered within the “ocean” of the C matrix. Previous research has unveiled an asynchronous lithiation/delithiation process in Si and C components within the Si/C composite anode.[^1^, ^11^] During the initial half of the charging process, lithiation predominantly occurs in Si, while delithiation only commences in the C material once it is fully lithiated during the discharging process. This phenomenon results in a distinctive occurrence where a “spot‐shaped” current forms around the Si “island” during the late stages of discharging when only the Si material is undergoing delithiation. Consequently, this may contribute to the entity of the internal impedance. A practical scenario associated with this concern is the potential shutdown of a cell phone equipped with a Si/C composite‐based battery, even when there is still available power. This is due to the significant impedance during the late stages of discharging, which hinders the delithiation of Si materials, leading to a rapid voltage reduction to the cutoff voltage and triggering the “shut down” signal. Solving this issue is significant for the wide application of Si/C composite anode for the next‐generation LIBs.

Considerable research efforts have been dedicated to exploring LIBs with Si/C composite anodes, focusing primarily on fundamental aspects like understanding lithiation/delithiation processes,^[^ ^12^ ^]^ mechanical volume changes,^[^ ^13^ ^]^ stress development,^[^ ^14^ ^]^ and the interplay between these processes.[^1^, ^15^] Researchers have employed advanced experimental methods, including in situ techniques,[^14^, ^16^] to achieve these objectives. Computational models have played a crucial role, evolving from pure electrochemical models^[12c]^ to fully coupled models.[^1^, ^17^] More and more details have been introduced into the multiphysics modeling framework, such as plasticity,^[^ ^18^ ^]^ crack formation and debonding,^[^ ^19^ ^]^ solid–electrolyte interphase formation,^[^ ^20^ ^]^ and more, enhancing the models to describe specific scenarios. Despite this extensive exploration, there appears to be a gap in the literature regarding the evolution of impedance inhomogeneities induced by the incorporation of Si materials into the C matrix of Si/C composite anodes. This knowledge gap poses a challenge to the widespread commercial adoption of this promising material in the next‐generation high‐energy‐density LIBs.

To bridge this gap, in the present study, we established an electrochemo‐mechanical‐coupled modeling framework aiming specifically at Si monoxide/graphite (SiO/Gr) composite anode materials (the Si and C elements exist in SiO and Gr, respectively) based on our previous modeling strategy.^[1b]^ This article is organized as follows: 1) first, five polarization components are introduced and analyzed based on the baseline case (5 wt% of SiO), among which two of them are proved to be dominant during the normal charging/discharging process: diffusion polarization in solid and activation overpotential; 2) then, the effects of SiO wt%, electrode thicknesses, SiO uneven distributions, and SiO distribution directions on the polarization evolution are discussed, especially about these two major polarization components; and 3) finally, the conclusions are summarized.

## 2 Results

The effects of SiO particles on the impedance evolution of the battery with SiO/Gr composite anode are first generally analyzed based on a SiO 5 wt% case (baseline). The details are as follows.

### 2.1 Definition of the Averaged Polarization Components

According to the previous study,^[^ ^21^ ^]^ the polarization of the battery can be influenced by three major subprocesses: activation of the electrochemical reactions, mass transport of species, and inadequate contact between various materials in the electrodes. The polarization associated with the activation of the electrochemical reactions is called the activation. The mass transport of species results in two types of polarizations, named diffusion polarization (due to the concentration gradients in both liquid and solid phases) and Ohmic potential drops (due to the insufficient ionic and electronic conductivities in the electrolyte and solid phase, respectively). The polarization stemming from the third subprocess is neglected in this study because the contact resistance between the current collector and the electrode is not included in the model and only the polarizations in anode part are interested. According to the aforementioned definition, five polarization components are analyzed in this study in the following sections (**Table** **1**).

**Table 1:** The averaged polarization component expressions.

Polarization components	Expressiona)	Equation number
Diffusion polarization in solid phase, *p*_ds_	$\frac{1}{i_{\text{tot}}}\int_{\Omega }i_{\text{loc}}(E_{\text{surf}}-E_{\text{ave}})dV$	(1)
Diffusion polarization in electrolyte, *p*_dl_	$-\frac{1}{i_{\text{tot}}}\int_{\Omega }\frac{2RT}{c_{l}F}\kappa_{c}\nabla c_{l}\cdot i_{l}dV$	(2)
Ohmic potential drop in solid phase, *p*_os_	$\frac{1}{i_{\text{tot}}}\int_{\Omega }\frac{i_{s}^{2}}{\kappa_{s}^{\text{eff}}}dV$	(3)
Ohmic potential drop in electrolyte, *p*_ol_	$\frac{1}{i_{\text{tot}}}\int_{\Omega }\frac{i_{l}^{2}}{\kappa_{l}^{\text{eff}}}dV$	(4)
Activation overpotential, *p*_ao_	$\frac{1}{i_{\text{tot}}}\int_{\Omega }i_{\text{loc}}(\varphi_{s}-\varphi_{l}-E_{\text{surf}})dV$	(5)

a) Here, $i_{\text{tot}}=\int_{\Omega }i_{\text{loc}}dV$ is the total current density and $\kappa_{c}=(1+t_{+})(1+\partial \ln f_{\pm }/\partial \ln c_{l})$ is the concentration conductivity. Within the electrode domain, Ω, if the parameters are not available, the corresponding integration is considered to be zero.

### 2.2 Typical Results Analysis

**Figure 1:**
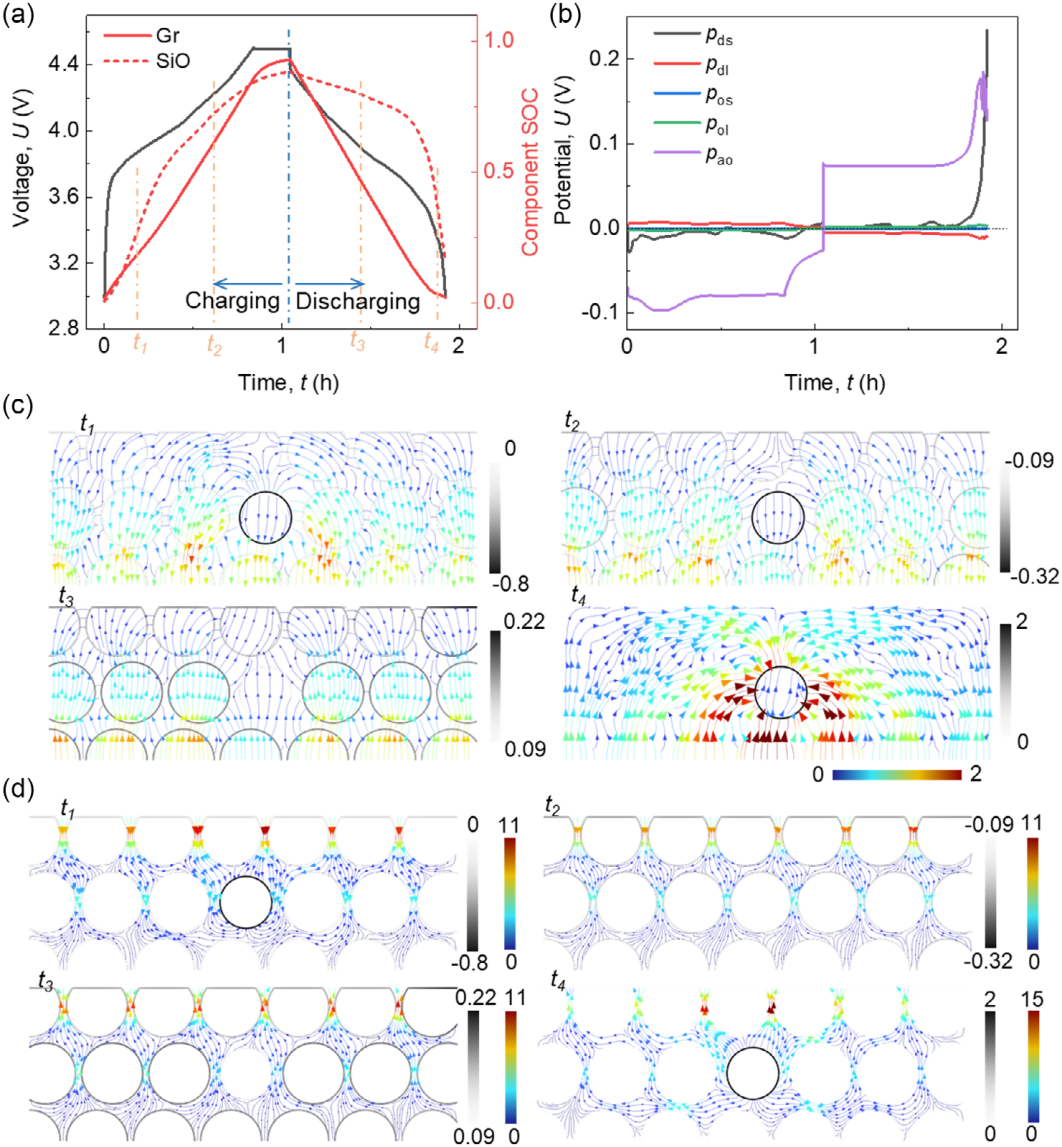
Computation results of the SiO/Gr composite anode (SiO ~5%) during the charging/discharging cycling process about a) voltage and component SOC profiles; b) averaged polarization components profiles (the definition of the components can be found in Table 1); the normalized current density (divided by 1C current density) distributions in c) solid phase and d) liquid phase considering the local current density at the particle and CBD + E interface at four selected time points (marked in (a)).

Under 1C current, the charging time is slightly larger than 1 h while the discharging time is smaller than 1 h (**Figure** **1**a). This indicates that the discharging capacity is smaller than the charging capacity, which is the issue mentioned in Introduction. A more detailed insight is provided by examining the state of charge (SOC) curves of the individual components. During the discharging process, the initial delithiation primarily occurs within the Gr component. It is only after Gr has undergone complete delithiation that the major delithiation of SiO commences. However, this SiO delithiation phase is exceedingly short, and the component SOC of SiO only decreases to 0.175, signifying the presence of residual Li ions stored within SiO particles. This observation also indirectly demonstrates the existence of extremely high impedance values at low SOC. When looking at the averaged polarization components (Figure 1b), two of them are found to be dominant: the diffusion polarization in the solid phase (*p*_ds_) and the activation overpotential (*p*_ao_). The other three components are neglectable compared to these two dominant factors. Therefore, the subsequent discussion will center on these pivotal polarization components. In the case of *p*_ds_, we observe a slight increase at the onset of charging, followed by a rapid increase at the end of discharging, ultimately reaching 0.235 V. At other times, the polarization value remains significantly lower. As for *p*_ao_, it exhibits relatively stable polarization values (≈0.08 V) during charging and the majority of the discharging process. Similar to *p*_ds_, there is a gradual increase at the initiation of charging and a sharp rise toward the conclusion of discharging, reaching 0.097 and 0.178 V, respectively. These notable increases in polarization values at the end of discharging align with the experimental findings (see Experimental Section). The current density distributions in the solid phase at time points *t*_1_ and *t*_4_ demonstrate the nonuniform flows, named as “spot shape current” in this study. Specifically, the current flows outward from SiO to Gr at *t*_1_ and inward to SiO from Gr at *t*_4_ (Figure 1c). In contrast, at time points *t*_2_ and *t*_3_, the current follows a predominant path along the thickness direction, a typical behavior observed in non‐composite electrodes. Notably, a relatively high local current value on the surface of SiO particles is observed at *t*_1_ and *t*_4_. The current density distribution within the electrolyte reflects a similar pattern (Figure 1d). These collective findings substantiate a consistent conclusion: the high impedance at the end of the discharging process primarily stems from diffusion polarization and activation overpotential within SiO particles.

Equation (1) clearly illustrates that the substantial diffusion polarization observed in SiO particles at the end of the discharging process arises primarily from the significant Li^+^ concentration gradient within the SiO particle. This gradient, in turn, results in a notable variation between the average potential and the surface potential (Figure S1 and S2, Supporting Information). This pronounced gradient within the SiO particle is primarily a consequence of the sequential discharging behavior of the Gr and SiO particles, as discussed earlier. During this process, SiO undergoes rapid delithiation within a short timeframe, accompanied by high local current density, ultimately giving rise to a substantial Li^+^ concentration gradient along the radial direction of the particle. Furthermore, according to Equation (5), the large polarization due to activation overpotential observed at the end of the discharging phase is attributed to the higher overpotential value at the SiO particle surface when compared to Gr particles (Figure S3, Supporting Information). This divergence in overpotential values can be attributed to the complex interplay and coordination of chemical potentials between SiO and Gr materials. This intricate interaction leads to an elevated overpotential value near the SiO particle surface toward the conclusion of the discharging process, thus facilitating the delithiation in SiO.

## 3 Discussion

### 3.1 Effects of SiO wt%

**Figure 2:**
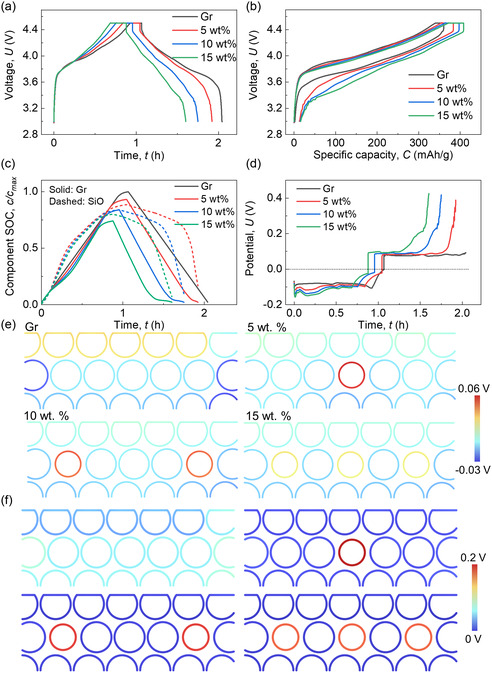
Computation results of the SiO/Gr composite anodes with various SiO wt% (0, 5, 10, and 15 wt%) during the charging/discharging cycling process about a) voltage versus time profiles; b) voltage versus capacity profiles; c) component SOCs of SiO and Gr materials; d) total polarization profiles; detailed distribution of e) the difference between surface potential and average potential, *E*_surf_ − *E*_ave_; and f) the overpotential, at time point *t*_4_ (defined in Figure S1, Supporting Information). Note: the anode thicknesses in these cases are the same.

Under the same charging/discharging protocol (see Experimental Section), it is observed that the cell with a higher SiO wt% in the anode exhibits shorter charging/discharging time (**Figure** **2**a). This is attributed to the fact that the cell with a higher SiO wt% undergoes charging and discharging at a higher current density (1C rate) due to its larger theoretical capacity. Consequently, the cell with a higher SiO wt% shows only a slightly larger value for specific capacities during the charging process, owing to the reduced charging time (Figure 2b). However, all cells with varying SiO wt% demonstrate the same residual capacity at the end of the discharging process, except for the cell with a pure Gr anode, which exhibits nearly 100% reversible capacity. Notably, despite the slightly higher specific charging capacity of the cell with a higher SiO wt%, an increase in SiO wt% leads to a decrease in the lithiation degree in both Gr and SiO particles (Figure 2c). This implies a reduction in active material utilization efficiency. Furthermore, cells with lower SiO wt% exhibit higher residual SOC values at the end of discharging in the SiO component. Since the delithiation processes in Gr are all thorough across various SiO wt%, it reveals that the less sufficient lithiation in SiO with smaller SiO wt% is the primary factor contributing to the smaller Coulombic efficiency. The polarization values in SiO and Gr components further demonstrate that larger polarizations mainly originate from the SiO material (Figure S4, Supporting Information).

The total polarization profiles reveal that the cell with larger SiO wt% shows larger average polarization values throughout the entire charging/discharging process, particularly showing significantly large values near the end of the discharging process (Figure 2d). This phenomenon accounts for the overall decrease in lithiation of both Gr and SiO particles with increasing SiO wt%. Examining the specific polarization components, the *p*_ds_ (Figure S4a, Supporting Information) shows extreme large values (≈0.3 V) at the end of discharging, while the *p*_ao_ (Figure S4b, Supporting Information) shows overall moderate values (≈±0.1 V) before the end of discharging. Thus, the total polarization profiles show a combination tendency as these two profiles. According to the equations in Table 1, the polarization value here is the specific integration upon the corresponding domain. When considering the distributions of the key factors at the end of the discharging (Figure 2e,f), it demonstrates that increasing the SiO wt% decreases the polarization in each SiO particle. However, the sum of the polarization in the same case is increased due to the increased quantity of SiO particles. Hence, there is a balance that needs to be considered during the design of SiO wt%. If only considering the outcome capacity delivery and neglecting the material wastes, increasing the SiO wt% with the same mass loading is suggested. However, increasing SiO wt% significantly increases the volume change (Figure S5a, Supporting Information), introducing substantial risks of mechanical issues resulting from residual volume expansion and associated stresses (Figure S5b–e, Supporting Information).

### 3.2 Effects of the Electrode Thickness/Capacity

**Figure 3:**
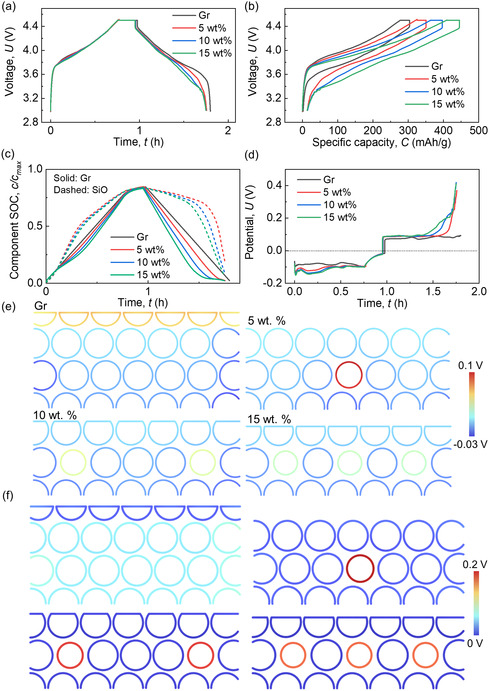
Computation results of the SiO/Gr composite anodes with various SiO wt% (0, 5, 10, and 15 wt%) during the charging/discharging cycling process about a) voltage versus time profiles; b) voltage versus capacity profiles; c) component SOCs of SiO and Gr materials; d) total polarization profiles; detailed distribution of e) the difference between surface potential and average potential, *E*_surf_ − *E*_ave_; and f) the overpotential, at time point *t*_4_ (defined in Figure S8, Supporting Information). Note: the theoretical maximum capacities in these cases are the same (anode thicknesses are different).

In the preceding section, uniform anode thicknesses are maintained across all cases with varying SiO wt%, resulting in different theoretical capacities. To isolate the effects from capacity, this section examines cells with varying SiO wt% but identical theoretical capacities, introducing the concept of an electrode thickness effect. Charging/discharging times of these cells are nearly identical when theoretical capacities are the same (**Figure** **3**a). However, cells with larger SiO wt% exhibit higher specific capacities due to reduced mass loadings (thicknesses) (Figure 3b). Consequently, decreasing the electrode thickness while keeping SiO wt% constant enhances specific capacity (Figure S6, Supporting Information). The overall SOC values in both SiO and Gr increase with 15 wt% SiO, while they decrease in cases with 0 and 5 wt% SiO, indicating improved lithiation with reduced electrode thickness (Figure 3c). Analysis of polarization values reveals a decrease in both 15 SiO and 5 wt% SiO cases when changing the thickness, contrary to overall component SOC behavior (Figure 3d). This decrease is mainly attributed to reduced diffusion polarization in the solid phase (Figure S7a, Supporting Information), while the activation overpotential‐induced polarization remains relatively unaffected by electrode thickness (Figure S7b, Supporting Information). If we look at the solid diffusion polarization in detailed component materials, it can be observed that the solid diffusion in SiO is still comparable in 5 wt% case to those in 10 and 15 wt% cases (Figure S7c, Supporting Information). The reason why the averaged diffusion polarization in solid phase (Gr + SiO) in 5 wt% case decreases is due to the smaller SiO wt% compared to the other two cases. The detailed distribution of the differences between surface potential and average potential supports this observation (Figure 3e and S8 and S9, Supporting Information). Regarding activation overpotential‐induced polarization, the detailed component (Figure S7d, Supporting Information) and distribution of overpotential (Figure 3f and S10, Supporting Information) exhibit similar behaviors to those in Section 3.1. Therefore, thinner electrodes, especially for those with high SiO wt%, are recommended. However, reducing electrode thickness leads to increased volume change and residual deformation after the discharging process (Figure S11a, Supporting Information), resulting in more challenging stress conditions (Figure S11b–e, Supporting Information) compared to corresponding thicker electrodes. To further demonstrate this conclusion, two more cases with the same SiO proportion of 10 wt% but different thicknesses (44 and 48 μm) were established (Figure S12, Supporting Information) to compare with the one with the 10 wt% of SiO and a thickness of 40 μm described earlier in Figure 3. The results confirm that thicker electrode produces less workable capacity (Figure S13a,b, Supporting Information), smaller material utilization efficiency (Figure S13c, Supporting Information)), larger impedance inhomogeneity (Figure S13d–f, Supporting Information), yet smaller deformation and stresses (Figure S14, Supporting Information) under the same C rate conditions (1C rate in this study).

### 3.3 Effects of the SiO Distribution Unevenness

**Figure 4:**
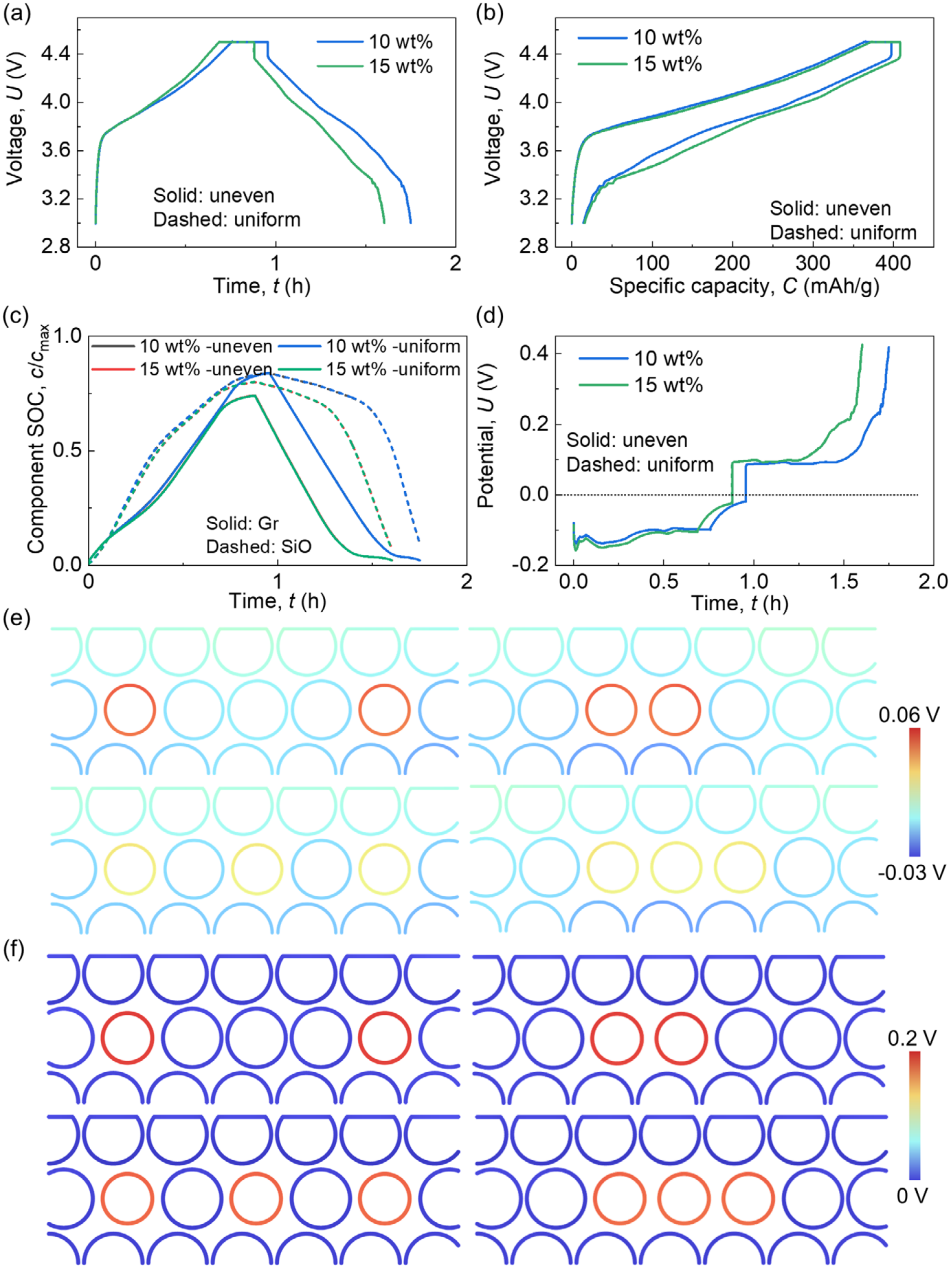
Computation results of the SiO/Gr composite anodes with two SiO wt% (10 and 15 wt%) considering two types of SiO distribution uniformities during the charging/discharging cycling process about a) voltage versus time profiles; b) voltage versus capacity profiles; c) component SOCs of SiO and Gr materials; d) total polarization profiles; detailed distribution of e) the difference between surface potential and average potential, *E*_surf_ − *E*_ave_; and f) the overpotential, at time point *t*_4_ (defined in Figure S1, Supporting Information).

The findings indicate that the nonuniform distribution of SiO along the in‐plane directions has negligible effects on battery electrochemical performance, as evidenced by consistent voltage behavior, capacity values, component SOC evolution, and polarization formation (**Figure** **4**a–d). The polarization components in SiO and Gr (Figure S15, Supporting Information) and the detailed distribution of the governing factors (Figure 4e,f, and S16 and S17, Supporting Information) further demonstrate this conclusion. Only slight differences are observed in minor polarization components, i.e., diffusion polarization in the electrolyte (*p*_dl_) and the Ohmic potential drops (*d*_os_ and *d*_ol_) (Figure S18, Supporting Information). However, these differences have minimal impact on the total polarization value, given the small magnitudes of these three components compared to the two major ones. In the mechanical aspect, the uneven distribution of SiO particles (SiO particle agglomeration) along the in‐plane direction results in a larger average volume change than uniform distributions for both 10 and 15 wt% cases (Figure S19a, Supporting Information). This effect is more pronounced with higher SiO wt%. Furthermore, uneven SiO distribution leads to significant stress concentrations within the SiO agglomeration and the surrounding area (Figure S19b,c, Supporting Information). Thus, although the uneven distribution of SiO particles does not influence electrochemical performance in a single cycle, it adversely affects mechanical behavior, increasing risks to cycling mechanical integrity.

### 3.4 Effects of the SiO Distribution Direction

**Figure 5:**
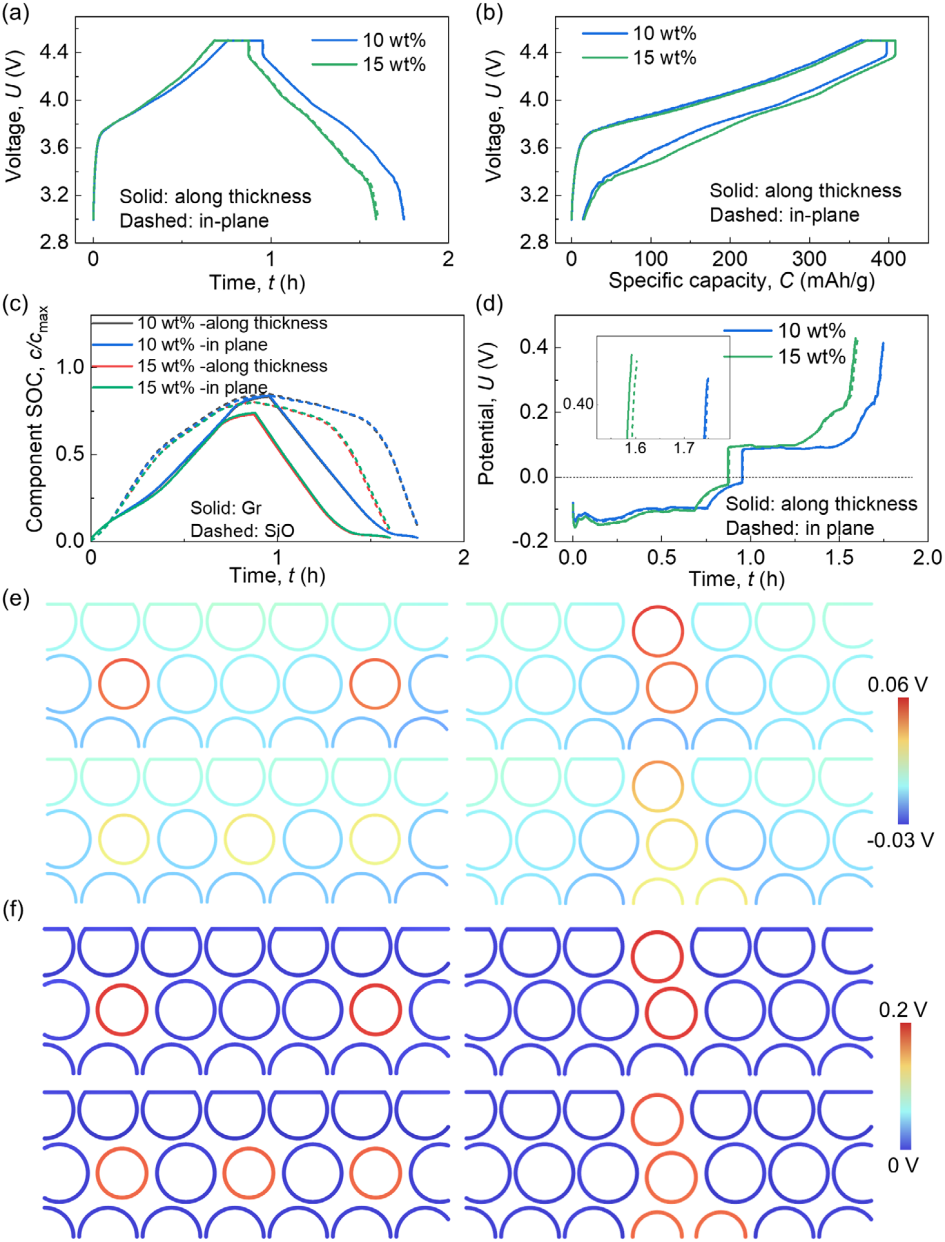
Computation results of the SiO/Gr composite anodes with two SiO wt% (10 and 15 wt%) considering two types of SiO distribution directions during the charging/discharging cycling process about a) voltage versus time profiles; b) voltage versus capacity profiles; c) component SOCs of SiO and Gr materials; d) total polarization profiles; detailed distribution of e) the difference between surface potential and average potential, *E*_surf_  − *E*_ave_; and f) the overpotential, at time point *t*_4_ (defined in Figure S1, Supporting Information).

Distributing SiO particles along the thickness direction results in a slightly higher charging voltage and a slightly lower discharging voltage compared to cases with SiO distribution along the in‐plane direction (**Figure** **5**a). This difference is minimal, leading to nearly overlapping specific capacity curves between cases with SiO distributed along the thickness and in‐plane directions (Figure 5b). The distribution of SiO along the thickness direction hinders the lithiation/delithiation process in Gr and the delithiation process in SiO, while slightly promoting the lithiation process in SiO, especially for the 10 wt% case (Figure 5c). Correspondingly, a relatively smaller polarization at the charging beginning of case 10 wt% with SiO distribution along thickness direction can be observed, while larger polarization values near the end of the discharging process are caused by the thickness direction distribution of SiO for both case 10 and case 15 wt% (Figure 5d). The polarization components show the same tendency (Figure S20, Supporting Information), supported by the detailed distribution of the governing factors (Figure 5e,f, and S21 and S22, Supporting Information). This indicates that larger values of the factors are induced in SiO near the end of the discharging process with SiO distribution along the thickness direction compared to distribution along the in‐plane direction. As for mechanical behaviors, a smaller average volume change is observed in the case with SiO distribution along the thickness direction compared to uniform SiO distribution for the 15 wt% case (Figure S19a, Supporting Information). For the 10 wt% case, distributing SiO along the thickness direction results in a smaller average volume change than distributing SiO unevenly along the in‐plane direction but is still larger than that of the uniform in‐plane distribution case. The stress conditions with SiO distribution along the thickness direction are worse than in‐plane distribution (both even and uneven) for both the 10 and 15 wt% cases (Figure S19b–d, Supporting Information).

## 4 Conclusion

The integration of Si‐based materials into conventional graphite anodes introduces complex electrochemical and mechanical interactions. Surprisingly, one aspect that has received relatively little attention from researchers is the impedance inhomogeneity, a critical factor that significantly undermines the discharging capacity of Si‐based LIBs. Thus, through the establishment of an electrochemo‐mechanical‐coupled model with detailed particle geometries on the anode side, this research systematically examined polarization components and their evolution during the charging/discharging process. Five representative polarization components that occur during the electrochemical cycling due to the subprocesses are introduced: two diffusion polarizations in the solid phase and liquid phase, two Ohmic potential drops in solid phase and liquid phase, and the activation overpotential. The typical behaviors of the polarizations during the charging/discharging cycling are analyzed based on the baseline case. Then, various factors influencing these dynamics, including SiO weight percentage (wt%), electrode thickness, and SiO distribution characteristics (distribution uniformity and direction), are methodically explored.

In conclusion, the following results can be summarized. 1) Out of the five polarization components, the diffusion polarization in the solid phase and the polarization induced by activation overpotential emerge as the two predominant factors. The primary source of these effects can be attributed to the presence of SiO particles. 2) Increasing the SiO wt% results in reduced polarization within individual SiO particles but concurrently leads to an increase in the overall average polarization of the cell. Achieving equilibrium among the resultant capacity, active material utilization rate, and Coulombic efficiency in SiO requires thoughtful consideration in SiO wt% design. Additionally, increasing SiO wt% significantly increases the volume change, introducing considerable risks of mechanical failures and, consequently, impacting the cell's durability. 3) A thinner electrode is advisable due to its superior performance, characterized by lower polarization values compared to thicker ones, particularly in cases with a high SiO wt%. However, it is essential to note that reducing electrode thickness introduces heightened volume change and residual deformation, thereby posing challenges in managing stress conditions. 4) SiO distributions, whether in‐plane uneven or along the thickness direction, do not impact solid diffusion and activation overpotential polarization. Trivial effects are observed in electrolyte diffusion and Ohmic potential drops. Therefore, SiO wt% is the primary factor influencing polarization, regardless of SiO particle distribution. In terms of mechanics, a uniform distribution of SiO particles is preferable due to a moderate condition of stress and strain.

The modeling insights gained from this study not only provide an efficient means of analyzing battery polarizations but also significantly contribute to a deeper understanding of the inhomogeneous evolution of these polarizations in Si/C composite anodes. These findings, in turn, offer valuable guidance for the strategic design of anodes in the pursuit of enhancing the performance and viability of next‐generation high‐energy‐density LIBs.

## 5 Experimental Section

5.1

5.1.1

##### Multiphysics Modeling Frameworks

An electrochemo–mechanical‐coupled model was established where two main physics were considered: electrochemical field and mechanical field. These two fields were coupled by transferring essential parameters with reasonable assumptions. The details are described in the following sections.

##### Multiphysics Modeling Frameworks: Electrochemical Field

The electrochemical behaviors were described by the sub‐models of a classical porous electrode theory (Newman's battery model) that are summarized in **Table** **2**.

**Table 2:** Newman's battery model.

Electrode level		Equation number
Current density in the liquid phase	$i_{l}=-\kappa_{l}^{\text{eff}}\left[\nabla \phi_{l}-\frac{2RT}{F}\left(1+\frac{d\ln f_{\pm }}{d\ln c_{l}}\right)\left(1-t_{+}\right)\nabla \ln c_{l}\right]$	(6)
Current density in solid phase	$i_{s}=-\kappa_{s}^{\text{eff}}\nabla \phi_{s}$	(7)
Li^+^ flux density in liquid phase	$J_{l}=-D_{l}^{\text{eff}}\nabla c_{l}+\frac{t_{+}}{F}i_{l}$	(8)
Charge conservation	$\nabla \cdot i_{l}=a_{s}i,\;\;\nabla \cdot i_{s}=-a_{s}i$	(9)
Mass conservation	$\varepsilon_{l}\frac{\partial c_{l}}{\partial t}=-\nabla \cdot J_{l}+\frac{a_{s}i}{F}$	(10)
Particle level		
Li‐ion intercalation/deintercalation	$\frac{\partial c}{\partial t}+\nabla \cdot J=0,\;\;J=\frac{D_{\text{eff}}}{RT}\left(FK\nabla c+\Omega c_{\max}\nabla \sigma_{h}\right)$	(11)
Particle–electrolyte interface		
Local current density	$i=i_{0}\left\{\exp\left[\frac{(1-\beta )F\eta }{RT}\right]-\exp\left(-\frac{\beta F\eta }{RT}\right)\right\}$	(12)
Exchange current density	$i_{0}=Fk_{c}^{\alpha_{a}}k_{a}^{\alpha_{c}}{\left(c_{\max}-c_{surf}\right)}^{\alpha_{a}}{\left(c_{surf}\right)}^{\alpha_{c}}{\left(c_{l}/c_{\text{l,ref}}\right)}^{\alpha_{a}}$	(13)

##### Multiphysics Modeling Frameworks: Mechanical Field

Both elastic and plastic behaviors were considered in the proposed model. For the elastic stage, the equilibrium equation was written by

(14) ∇⋅T+B=0

where **T = C:E** is the nominal stress tensor (**C** is the stiffness matrix and **E** is elastic strain matrix) and **B** is the body force which equals zero here. For the plastic stage, the von Mises law was used as the yield law for all the materials. In particular, a perfect plasticity model was applied for SiO particles with an initial yield strength related to the Li‐ion concentration, while a linear plasticity model (tangential modulus is used as in **Table** **3**) was introduced for the carbon binder domain + eletrolyte (CBD). Here, the Gr particles were only considered in elastic stage.

**Table 3:** Input parameters and values in the established multiphysics model.

Parameter	Symbol	Value
Electrode level		
RVE width	$W_{\text{RVE}}$	112 μm (estimated)
Anode thickness (single coating)	$L_{\text{an}}$	40 μm (measured)
Separator thickness	$L_{\text{sep}}$	10 μm (measured)
Cathode thickness (single coating)	$L_{\text{ca}}$	48 μm (measured)
Transference number^[^ ^22^ ^]^	$t_{+}$	*t*_*+*_(*c*_l_)
Transfer coefficient^[^ ^22^ ^]^	$\alpha_{a}$ $\alpha_{c}$	0.5
Diffusion coefficient in electrolyte^[^ ^22^ ^]^	$D_{e}$	$D_{e}(c_{l})$
Modulus of CBD + E domain	*E*_CBD_	2 GPa (estimated according to ref. [23])
Tangential modulus of CBD + E domain	*E*_t_CBD_	100 MPa (estimated)
Modulus of separator	*E*_sep_	200 MPa (estimated)
Modulus of cathode^[^ ^24^ ^]^	*E*_ca_	500 MPa (modified)
Particle level		
Graphite particle radius	*R*_Gr_	8 μm (measured)
SiO particle radius	*R*_SiO_	6.8 μm (measured)
Electrical conductivity of Gr^[^ ^25^ ^]^	$\kappa_{s}^{\text{cathode}}$	100 S m^−1^
Electrical conductivity of SiO^[^ ^26^ ^]^	$\kappa_{s}^{\text{anode}}$	1 S m^−1^
Partial molar volume of SiO^[^ ^27^ ^]^	Ω_SiO_	4.625 × 10^−6^ m^3^ mol^−1^
Partial molar volume of Gr^[^ ^28^ ^]^	Ω_Gr_	3.17 × 10^−6^ m^3^ mol^−1^
Modulus of SiO^[^ ^29^ ^]^	*E*_SiO_	*E*_SiO_ (*c*_SiO_)
Modulus of Gr^[^ ^28^ ^]^	*E*_Gr_	3.85 + 16.446*x* GPa (calculated)
Maximum Li concentration in SiO^[^ ^26^ ^]^	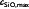	128 000 mol m^−3^
Maximum Li concentration in Gr^[^ ^30^ ^]^	*c*_Gr, max_	31 507 mol m^−3^
Diffusion coefficient in SiO^[^ ^31^ ^]^	*D*_0,Si_	1.5 × 10^−14^ m^2^ s^−1^
Diffusion coefficient in Gr^[^ ^32^ ^]^	*D*_0,C_	1.4 × 10^−13^ m^2^ s^−1^
Yield stress of SiO^[18b]^	*σ*_*y*, SiO_	$\sigma_{y\text{,SiO}}^{0}-0.4(c/c_{\max})\;\text{GPa}$ (modified)
Initial yield stress of SiO^[18b]^	$\sigma_{y,\text{SiO}}^{0}$	1 GPa (modified)

The total deformation gradient of the active particles **F** could be written out according to the multiplicative decomposition law as

(15) $$F=F_{e}\cdot F_{p}\cdot F_{l}$$

where the subscripts “**e**,” “**p**,” and “**l**” represent elastic, plastic, and lithiation induced, respectively. The elastic and plastic deformation were calculated by the elastic and plastic models mentioned earlier, respectively, while the lithiation‐induced volumetric deformation, **F**_**l**_, was related to the Li^+^ concentration in the solid phase, *c*, calculated from the electrochemical model. The lithiation‐induced deformation was assumed to be isotropic in this study with an expression as $F_{l}=\frac{\Omega }{3}\Delta c$.

##### Multiphysics Modeling Frameworks: Coupling Strategy

The electrochemical field and the mechanical field were coupled by transferring the hydrostatic stress σ_h_ and the Li^+^ concentration *c* between the mechanical model and the diffusion model as Equation (11) and (14) indicate. The hydrostatic stress, *σ*_h_, was defined as the mean value of three principal stresses, $\frac{\left(\sigma_{1}+\sigma_{2}+\sigma_{3}\right)}{3}$, which influenced the diffusion process within the particles according to the second term of Equation (11), named as the stress‐gradient‐driven diffusion. Contrarily, Li^+^ diffusion within the active particles triggered volume change (as $F_{l}=\frac{\Omega }{3}\Delta c$), subsequently resulting in the stress evolution within and among particles and the CBD.

In the present study, several assumptions were employed when coupling the models. First, the stress‐induced overpotential change ($\Omega \sigma_{h}/F$) was omitted due to its significantly smaller magnitude, approximately two orders of magnitude lower than the observed overpotential range in the target scenario. Second, the mechanical strain was not transferred to the electrochemical field to prevent any pressure effects on the electrolyte flows. Finally, the impact of volumetric change on the diffusion process was considered by introducing a coefficient in Equation (11) as *V*_1_/*V*_0_, where *V*_0_ represents the initial volume and *V*_1_ represents the expanded volume, respectively.

##### Implementation in Finite Element Computation Platform

**Figure 6:**
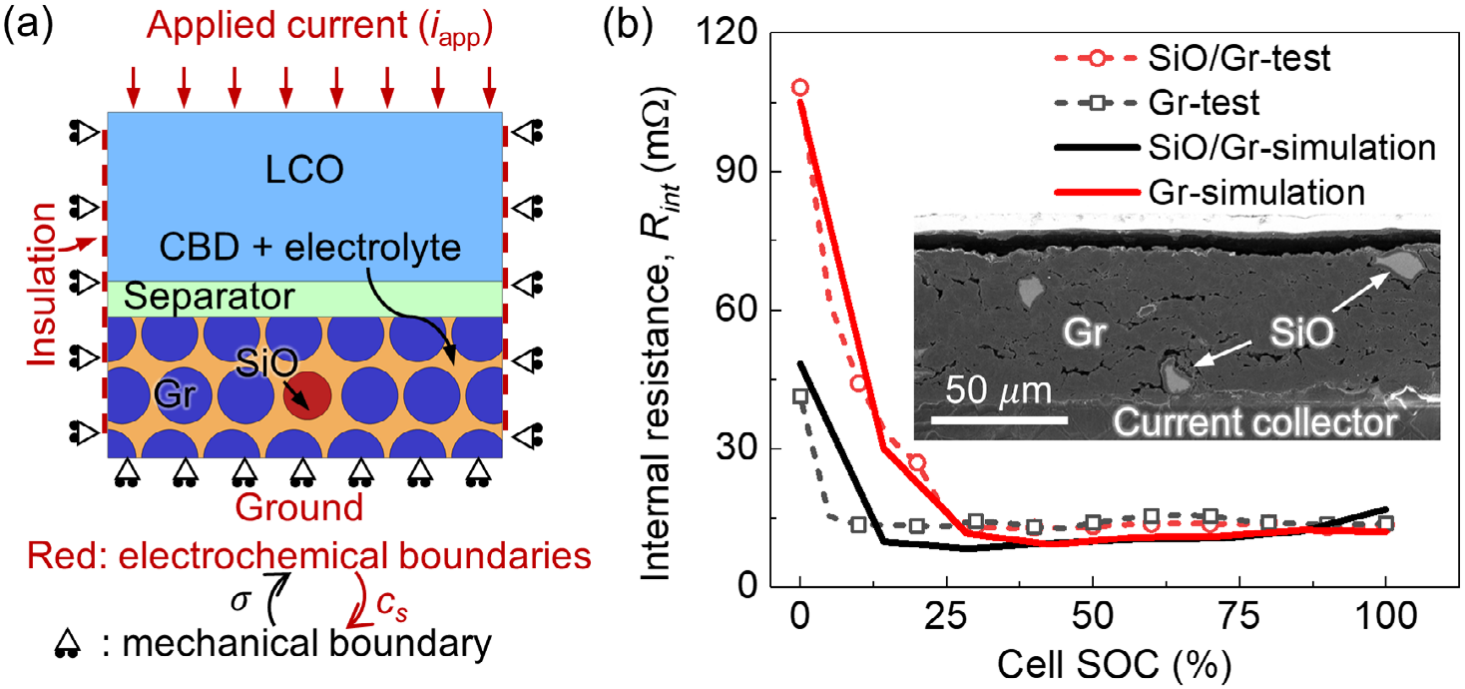
a) The illustration of the RVE model with coupling strategy and the boundary conditions; b) the comparison between the experimental results and the computational data of the dynamic impedance changes of the cells with pure Gr anode and SiO/Gr composite anode, respectively. The inset is the scanning electron microscope image of the SiO/Gr composite anode, showing the discrete distribution of the SiO particles within the Gr matrix.

A 2D representative volume element (RVE), incorporating detailed particle geometries in the anode layer, a separator layer, and a homogenized cathode layer, was extracted from a full‐cell configuration. This RVE was designated as the target for the subsequent numerical investigations (**Figure** **6**). The anode layer was composed of active particles (SiO and Gr), CBD, and electrolytes. The CBD and electrolyte were treated as a single domain (named as CBD + E), accounting for ≈36% of the total volume in all the models considered in this study. Here, the particles were considered as simplified spherical shapes with same diameters (see Table 3). The particles were assumed to be wrapped by the CBD + E domain, which provided both the electron and ion pathways. The CBD comprised ≈10% of the total volume of the anode in all the models considered in this study (the CBD proportion in the CBD + E domain is uniformly distributed and set as 0.3). The distribution of the SiO particles was manually determined according to the corresponding SiO wt%. The cathode layer was a homogeneous domain using lithium cobalt oxide (LCO) as the material in this study. The current collectors for both anode and cathode were not considered.

For electrochemical boundary conditions, the bottom surface of the anode layer was connected to the ground (0 V), while the top surface of the cathode layer was applied with a current density *i*_app_, for all the simulation cases (Figure 6a). The electrochemical loading was the charging–discharging cycling following this protocol: the model was employed by a 1C constant current charging first, followed by a constant voltage charging with cutoff current of 0.1C, then discharged by 1C constant current to the cutoff voltage of 3 V. The left and right sides are electrically insulated. The initial values of the Li^+^ concentration within the active particles were automatically distributed by the initiation process implemented in the COMSOL software. The software could automatically calculate and establish the initial conditions by providing it with the initial voltage and total capacity of the cell. For the mechanical boundaries, a roller constraint was defined for the bottom, right, and left sides while the top surface was set as free. (Figure 6a) The initial deformation and deformation gradient fields were set as zero.

##### Dynamic Impedance Test

A laboratory‐level cell, provided by Zhuhai CosMX Battery Co., Ltd., was employed to assess the dynamic impedance. This cell consisted of a SiO (5 wt%)/Gr composite anode and an LCO cathode with a nominal capacity of 800 mAh. The test procedure involved the following steps: initially, the cell was discharged with a constant current of 0.75C to 3 V, and subsequently the cell was charged using a constant current of 1C to 4.5 V, maintaining the voltage until the current decreased to 0.25 C (constant voltage charging). The cell was allowed to rest for 2 h. Following this, the cycling process was initiated: discharge with a constant current of 0.5C for 6 min, followed by a rest period of 35 min. These two steps were repeated until the voltage reached 2.8 V. The test was conducted at a temperature of 25 °C, and the dynamic impedance $R_{\mathrm{int}}(\text{SOC})$ was calculated using the following formula:

(16) $$R_{\mathrm{int}}(\text{SOC})=\Delta V(\text{SOC})/i_{\text{app}}$$

where $\Delta V(\text{SOC})=V_{2}(\text{SOC})-V_{1}(\text{SOC})$ is the difference between the voltage at the end of 35 min rest ($V_{2}(\text{SOC})$) and the voltage at the end of 0.5C constant current charging ($V_{1}(\text{SOC})$) for each SOC, and *i*_app_ is the applied current which is 0.5 C (400 mAh) in this study.

Before performing the fundamental analysis and parametric studies using the computational model, the results from the experiment and the numerical model were compared in terms of the dynamic impedances according to the testing method described earlier (Figure 6b). The results showed a good match between the test and the simulation. It indicated that both pure Gr anode and SiO/Gr composite anode exhibited relatively high impedance values at the low SOCs (particularly close to 0), while the addition of SiO resulted in an even higher value (about 2.65 times higher). In addition, the cell with SiO/Gr anode showed a wider range of high impedance values (SOC 0–0.25) than that of the cell with pure Gr anode (SOC 0–0.12).

##### Parametric Setups of Numerical Computation

**Figure 7:**
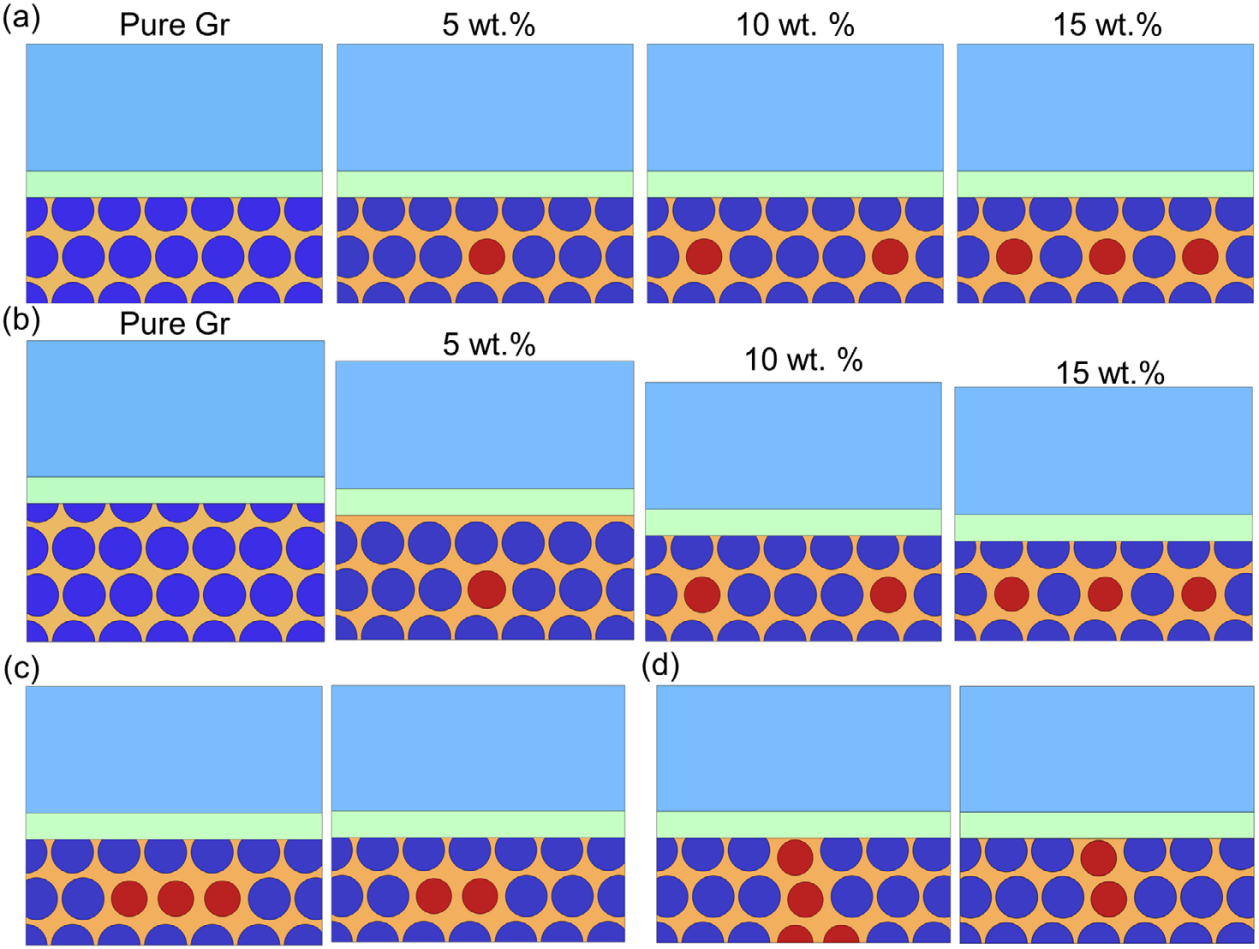
RVE models with various SiO wt% in anodes in a configuration of a) same thicknesses but different capacities; b) same capacities but different thicknesses; c) RVE models with SiO particles uneven distribution; and d) RVE models with SiO particles distributed along the thickness direction. The theoretical N/P ratio values for each case can be found in Table S1, Supporting Information.

In this study, the effects of the weight percentage of the SiO particle, the anode thickness/capacity, the distribution uniformity of SiO particles, and the distribution direction of the SiO particles on the impedance evolution were considered (**Figure** **7**). For the SiO wt% effect, three SiO wt% (5, 10, and 15 wt%) with a pure Gr case (SiO wt% = 0) were studied (Figure 7a). In this scenario, the thickness of the anode was the same among all the cases using the thickness value of the SiO 10 wt% case. Thus, the theoretical capacities of various models were different. To eliminate possible confusion, models with the same capacity and SiO wt% were conducted (Figure 7b). The model with lower SiO wt% thus showed a thicker anode. In these two scenarios, the SiO particles were uniformly distributed along the in‐plane direction. Based on our previous studies,[^1^, ^12^] it was demonstrated that the SiO location significantly influenced cell performance. Consequently, in the current work, the effects of SiO locations were also considered. Two representative scenarios were devised: SiO uneven distribution (agglomeration) (Figure 7c) and SiO distribution along thickness direction (Figure 7d). Here, we defined the uneven distribution as follows: the SiO particles are closely neighbored with each other, forming an agglomeration structure within the Gr matrix rather than being isolated. For each scenario, two SiO wt% (10 and 15 wt%) were considered in a same‐thickness manner.

All the aforementioned parametric cases were applied with the same charging–discharging protocol mentioned earlier. All the models in this study share the same material properties for SiO, Gr, electrolyte, and binders (Table 3).

## Nomenclature

**Table:** 

*c*	Li‐ion concentration in solid phase	$i_{s}$	Current density in solid phase	
J	Li‐ion flux	$\kappa_{s}^{\text{eff}}$	The effective electrical conductivity of solid phase	
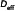	Effective Li‐ion diffusion coefficient	$\phi_{s}$	Potential in solid phase
*R*	Gas constant	$J_{l}$	Li‐ion flux in the electrolyte
*T*	Temperature	$\varepsilon_{e}$	The volume fraction of electrolyte
*F*	Faraday constant	$a_{s}$	Effective surface area per unit electrode volume
*K*	Rate constant	*i*	Intercalation reaction current density
*Ω*	Partial molar volume	*β*	Cathodic symmetric factor
$c_{\max}$	Maximum Li^+^ concentration	*η*	Over potential
$\sigma_{h}$	Hydrostatic stress	$i_{0}$	Exchange current density
*D*_0_	Initial Li‐ion diffusion coefficient	$E_{\text{ref}}$	Equilibrium potential of the active materials
$i_{l}$	Current density in electrolyte	$k_{a}$	The reaction rate constant for anode
$\kappa_{l}^{\text{eff}}$	The effective electrical conductivity of the electrolyte	$k_{c}$	The reaction rate constant for cathode
$\phi_{l}$	Potential in electrolyte		
$f_{\pm }$	Electrolyte activity coefficient		
$c_{l}$	Li^+^ concentration in electrolyte		

## Conflict of Interest

The authors declare no conflict of interest.

## Author Contributions

X.G.: Methodology (lead), Data Curation (lead), Formal Analysis (lead), Writing—Original Draft (lead); J.X.: Conceptualization (lead); Supervision (lead); Writing—Review and Editing (lead).

## Supporting information

Supplementary Material

## Data Availability

The data that support the findings of this study are available in the supplementary material of this article.
