# Supplementary material for: Impedance Inhomogeneity in SiO/Gr Composite Anode
Source: Small Sci. 2024 Jun 14;4(7):2300291. doi: 10.1002/smsc.202300291 (PMC11935037; doi:10.1002/smsc.202300291)
Supplement: Supplementary file 1 — Supplementary Material [file SMSC-4-2300291-s001.pdf]

Supplementary Information

Impedance inhomogeneity in SiO/Gr composite anode

Xiang Gao, Jun Xu*

Xiang Gao, Jun Xu*

Department of Mechanical Engineering, University of Delaware, Newark, DE 19716 USA;

Vehicle Energy & Safety Laboratory (VESL), University of Delaware, Newark, DE 19716 USA

Email: junxu@udel.edu

*Correspondence should be sent to Prof. Jun Xu. Email: junxu@udel.edu

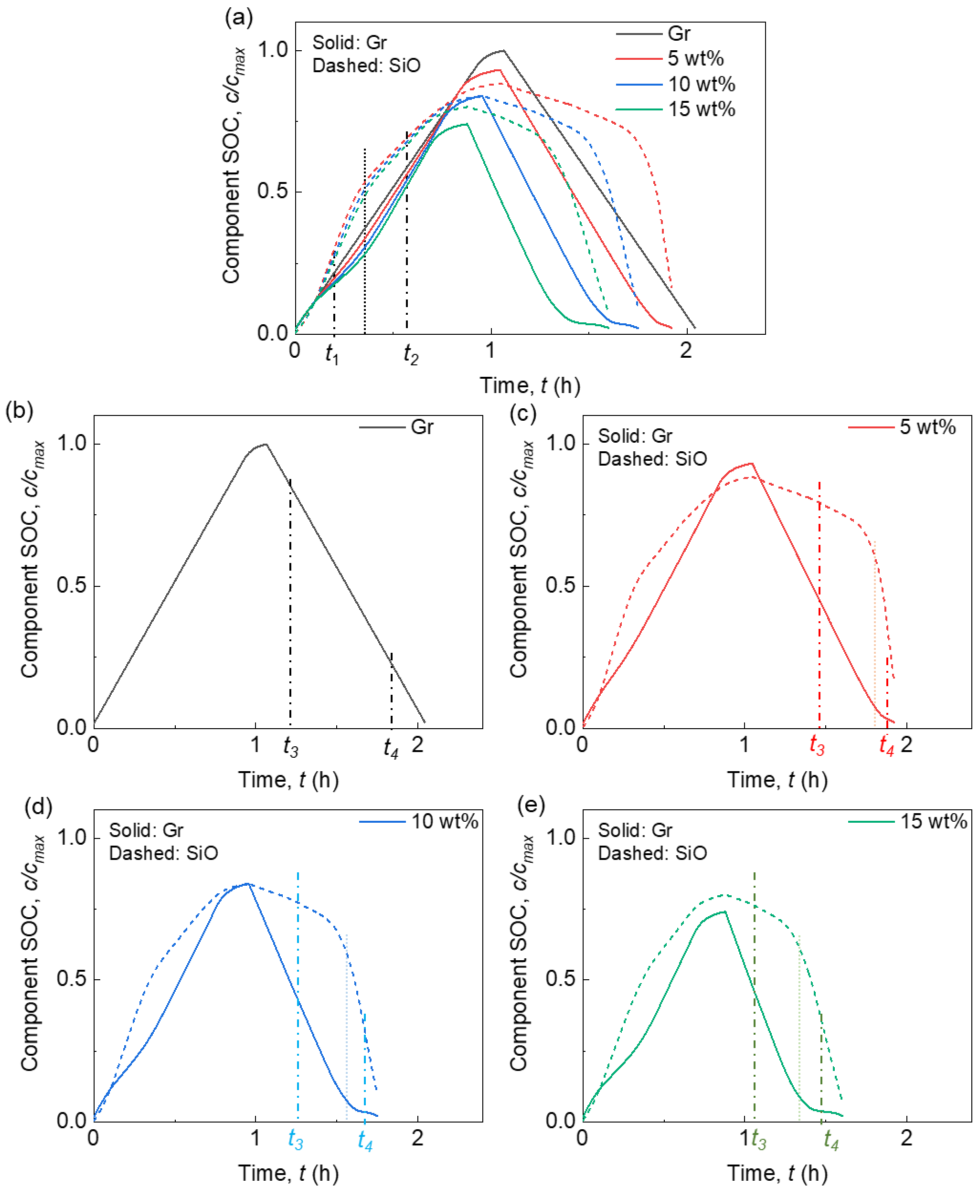

Figure S1. The definition of the selected time points for the cell with various SiO wt. %s under the condition of same electrode thickness. (a) The two time points in the charging process are the same among all the cases as t_1 = 900 s and t_2 = 2044 s; while the two time points in the discharging process are different for different cases as (b) pure Gr (t_3 = 4233 s and t_4 = 6739 s), (c) 5 wt. % SiO (t_3 = 5133 s and t_4 = 6760 s), (d) 10 wt. % SiO (t_3 = 4485 s and t_4 = 6055 s), and (e) 15 wt. % SiO (t_3 = 3844 s and t_4 = 5342 s).

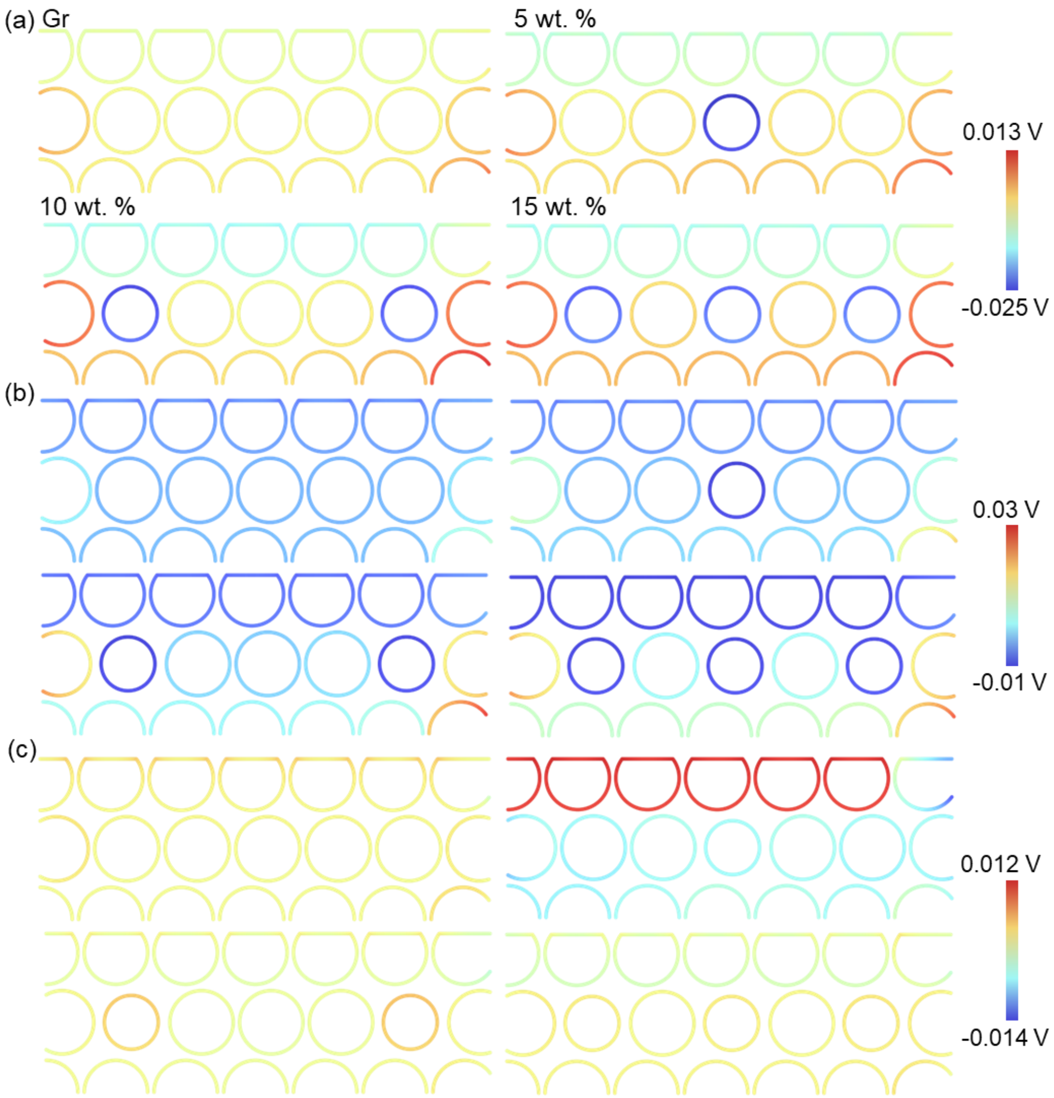

Figure S2. The detailed distribution of the differences between surface potential and average potential, $E_{surf} - E_{ave}$, of the SiO/Gr composite anodes with various SiO wt. %s (0 wt.%, 5 wt. %, 10 wt. %, and 15 wt. %) during the charging/discharging cycling process at time points (a) t_1, (b) t_2, and (c) t_3 (defined in Figure S1). Note: the anode thicknesses in these cases are the same.

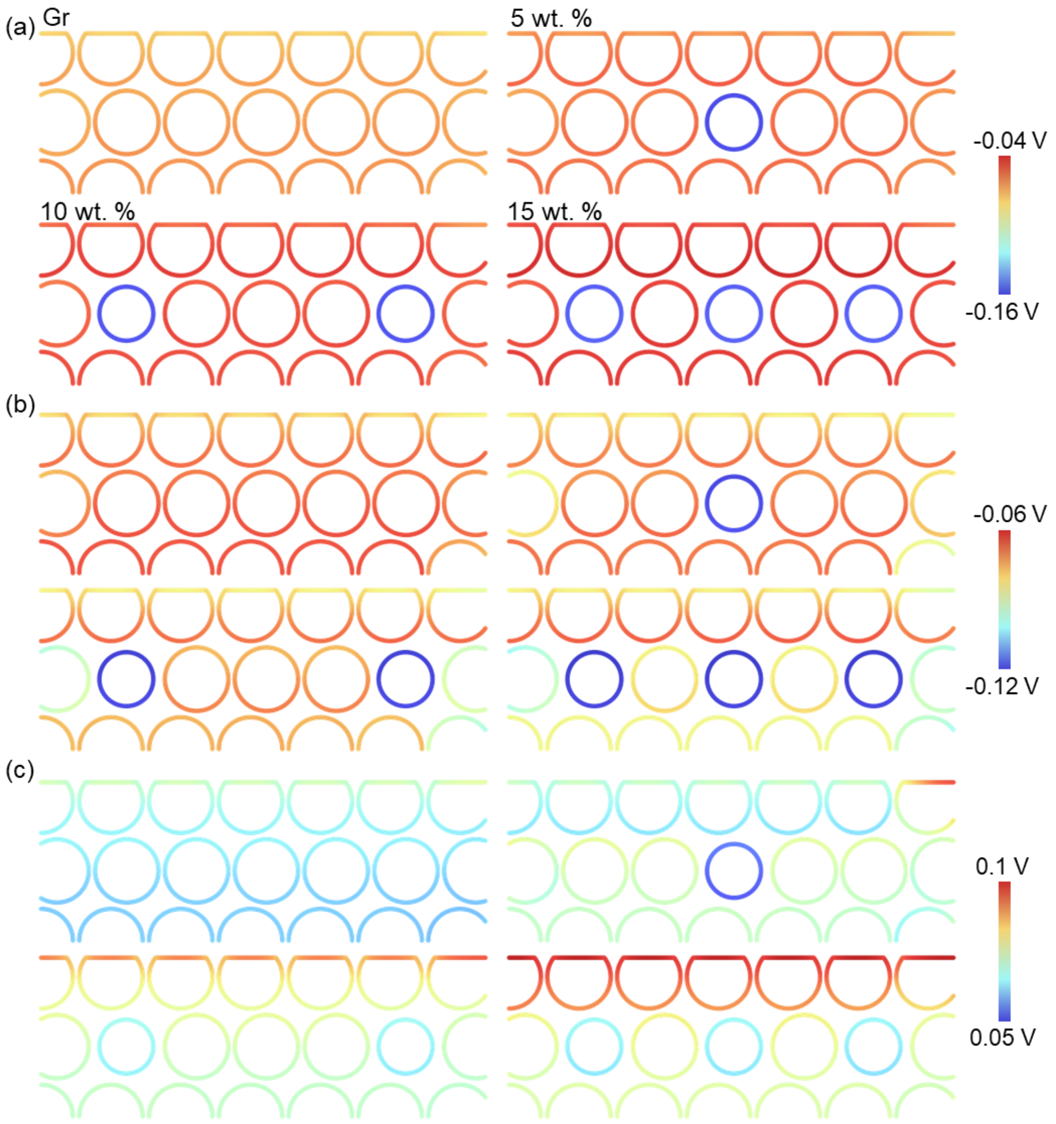

Figure S3 The detailed distribution of the difference between the overpotentials of the SiO/Gr composite anodes with various SiO wt. %s (0 wt.%, 5 wt. %, 10 wt. %, and 15 wt. %) during the charging/discharging cycling process at time points (a) t_1, (b) t_2, and (c) t_3 (defined in Figure S1). Note: the anode thicknesses in these cases are the same.

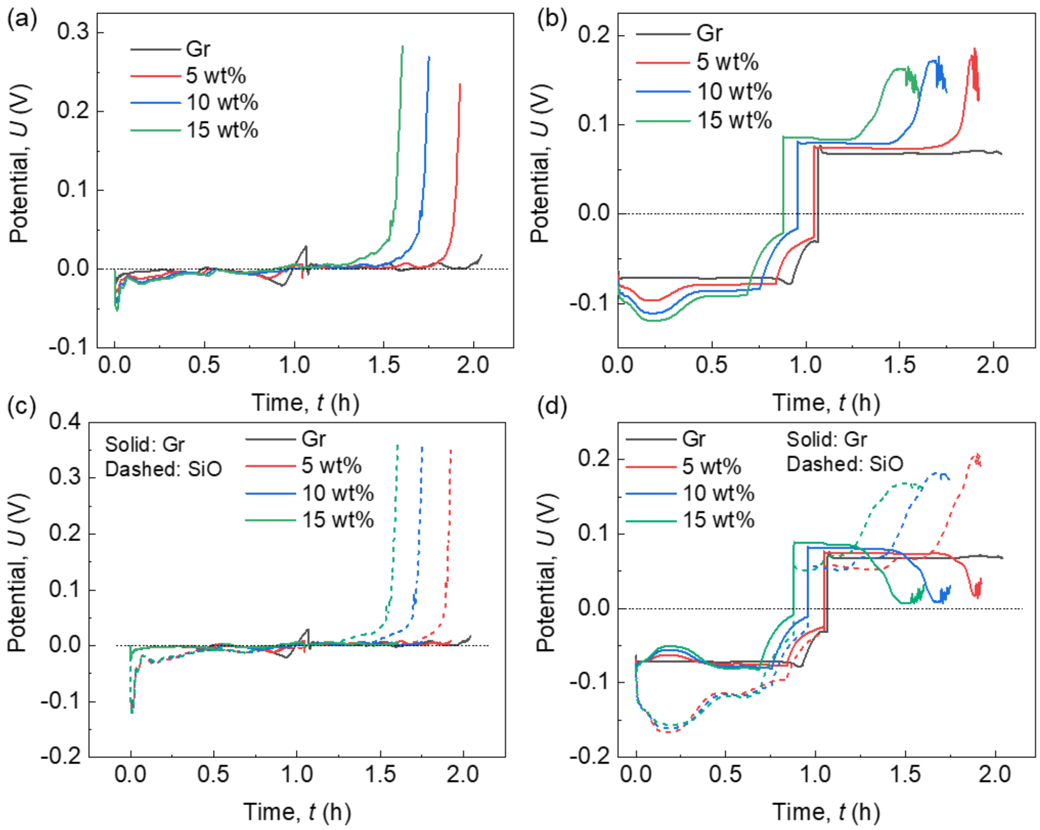

Figure S4. Computation results of the SiO/Gr composite anodes with various SiO wt. %s (0 wt.%, 5 wt. %, 10 wt. %, and 15 wt. %) during the charging/discharging cycling process about the polarization components of (a) diffusion polarization in solid phase and (b) activation overpotential. The polarization profiles in component materials (SiO and Gr) for (c) diffusion polarization in solid phase and (d) activation overpotential. Note: the anode thicknesses in these cases are the same.

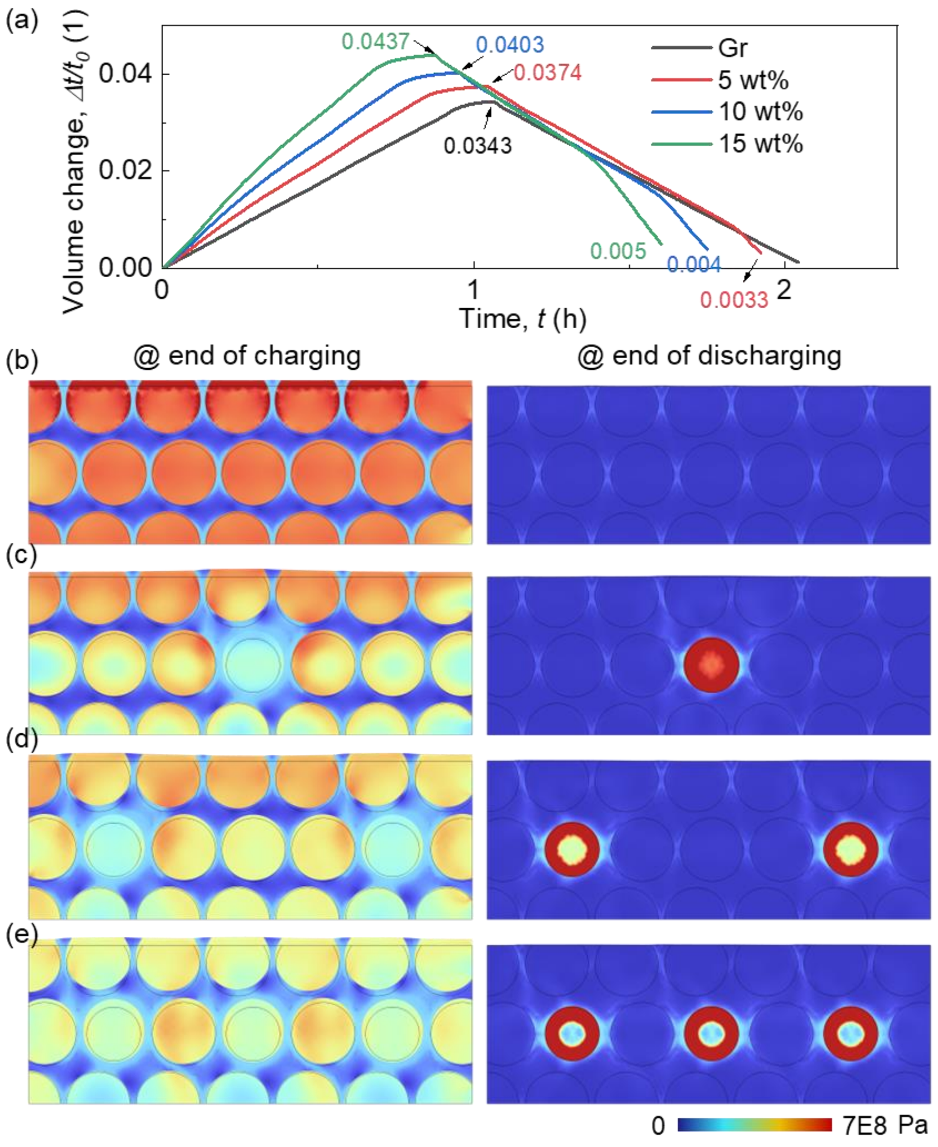

Figure S5. Computation results of the SiO/Gr composite anodes with various SiO wt. %s during the charging/discharging cycling process about (a) the overall volume changes with inset numbers indicating the ultimate values and residual values; the detailed Von Mises stress distributions at the end of charging and discharging processes of the cells with (b) 0 wt.%, (c) 5 wt. %, (d) 10 wt. %, and (e) 15 wt. %. Note: the anode thicknesses in these cases are the same.

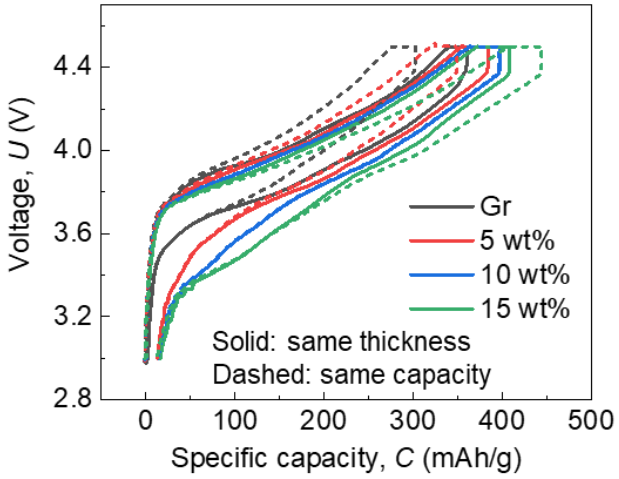

Figure S6. Comparison of the voltage vs. specific capacity profiles of the cells with various SiO wt. %s between the conditions with the same electrode thicknesses and the same theoretical maximum capacities.

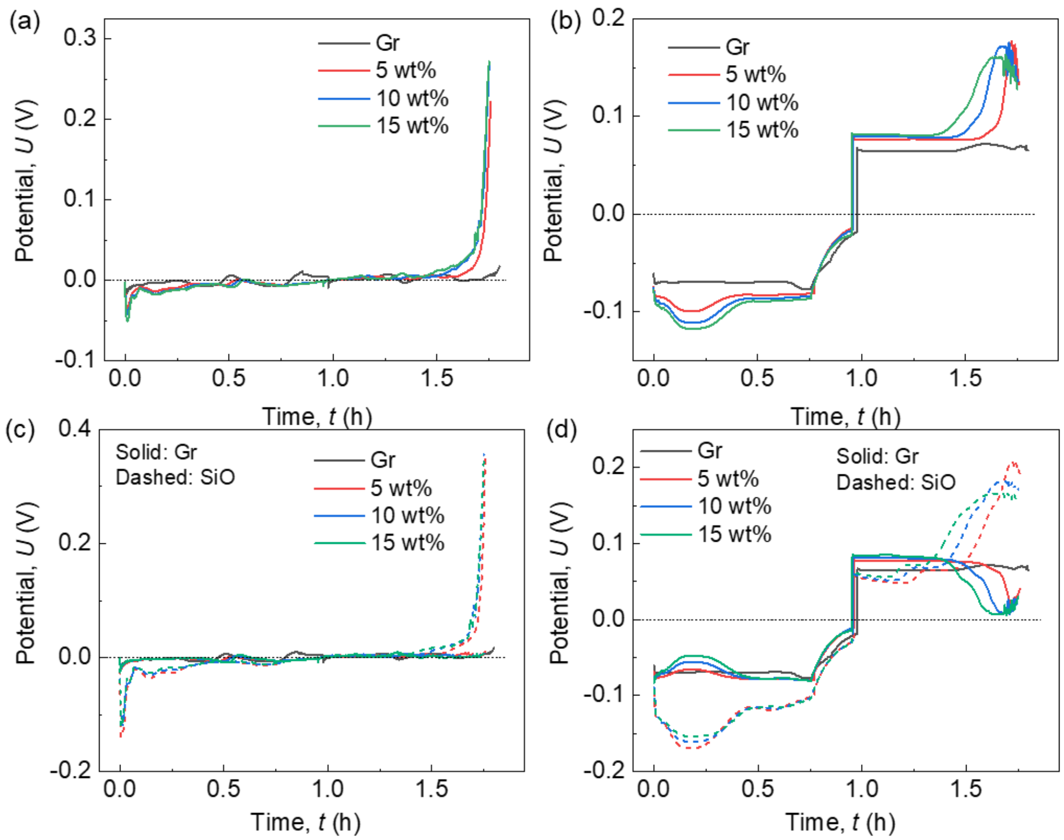

Figure S7. Computation results of the SiO/Gr composite anodes with various SiO wt. %s (0 wt.%, 5 wt. %, 10 wt. %, and 15 wt. %) during the charging/discharging cycling process about the polarization components of (a) diffusion polarization in solid phase and (b) activation overpotential. The polarization profiles in component materials (SiO and Gr) for (c) diffusion polarization in solid phase and (d) activation overpotential. Note: the anode theoretical maximum capacities in these cases are the same.

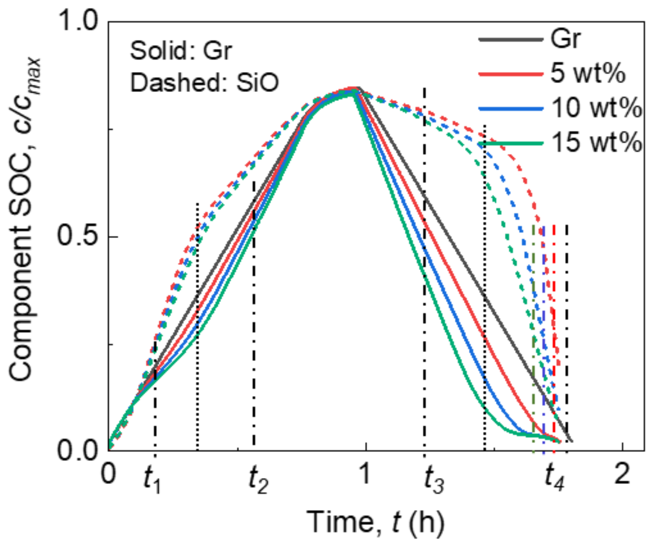

Figure S8. The definition of the selected time points for the cell with various SiO wt. %s under the condition of same electrode theoretical maximum capacities: The two time points in the charging process and one point in the discharging process are the same among all the cases as t_1 = 900 s, t_2 = 2044 s, and t_3 = 4406 s; while the last time point in the discharging process are different for different cases as pure Gr (t_4 = 6379 s), 5 wt. % SiO (t_4 = 6220 s), 10 wt. % SiO (t_4 = 6012 s), and 15 wt. % SiO (t_4 = 5890 s).

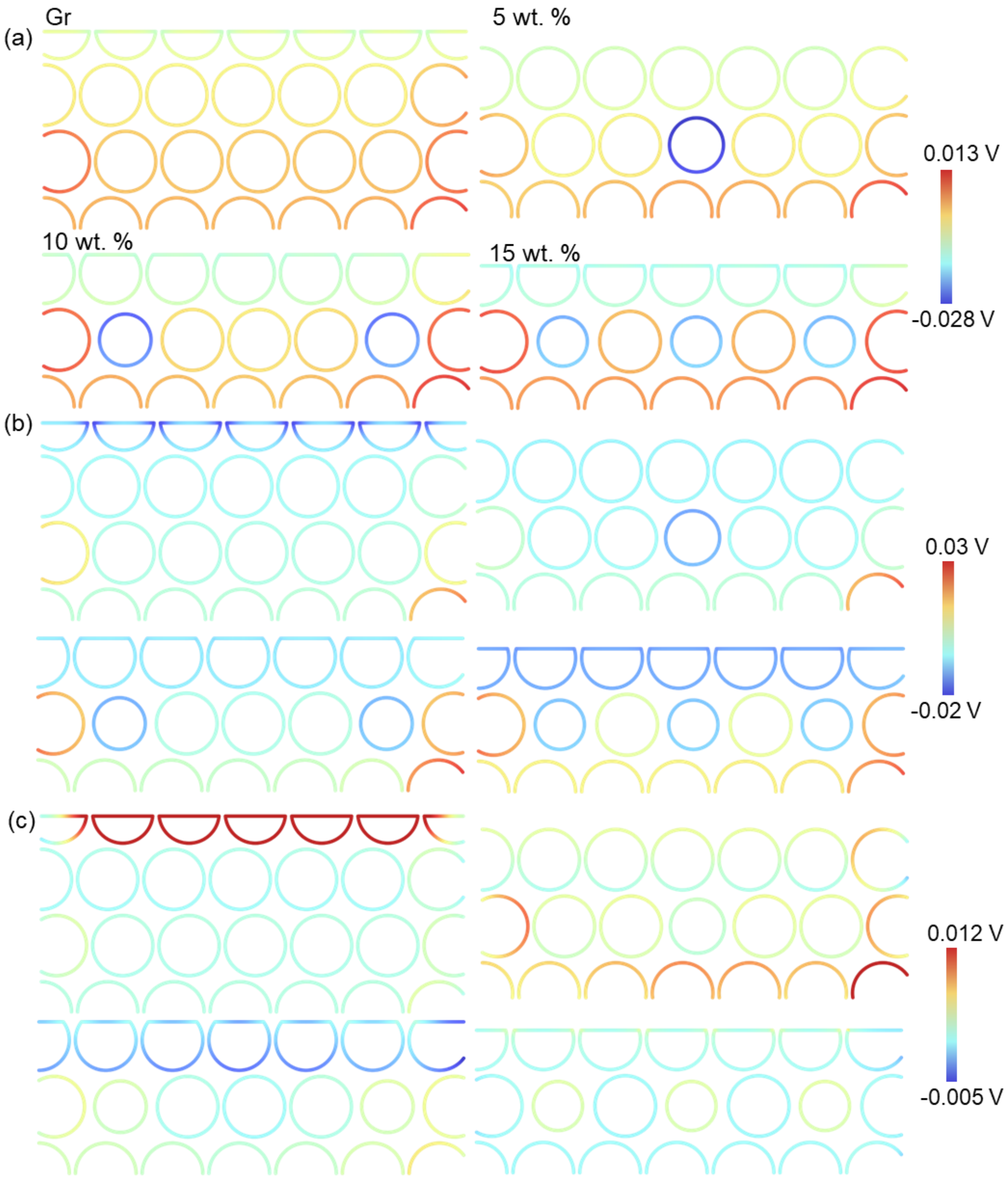

Figure S9. The detailed distribution of the differences between surface potential and average potential, $E_{surf} - E_{ave}$, of the SiO/Gr composite anodes with various SiO wt. %s (0 wt.%, 5 wt. %, 10 wt. %, and 15 wt. %) during the charging/discharging cycling process at time points (a) t_1, (b) t_2, and (c) t_3 (defined in Figure S8). Note: the anode theoretical maximum capacities in these cases are the same.

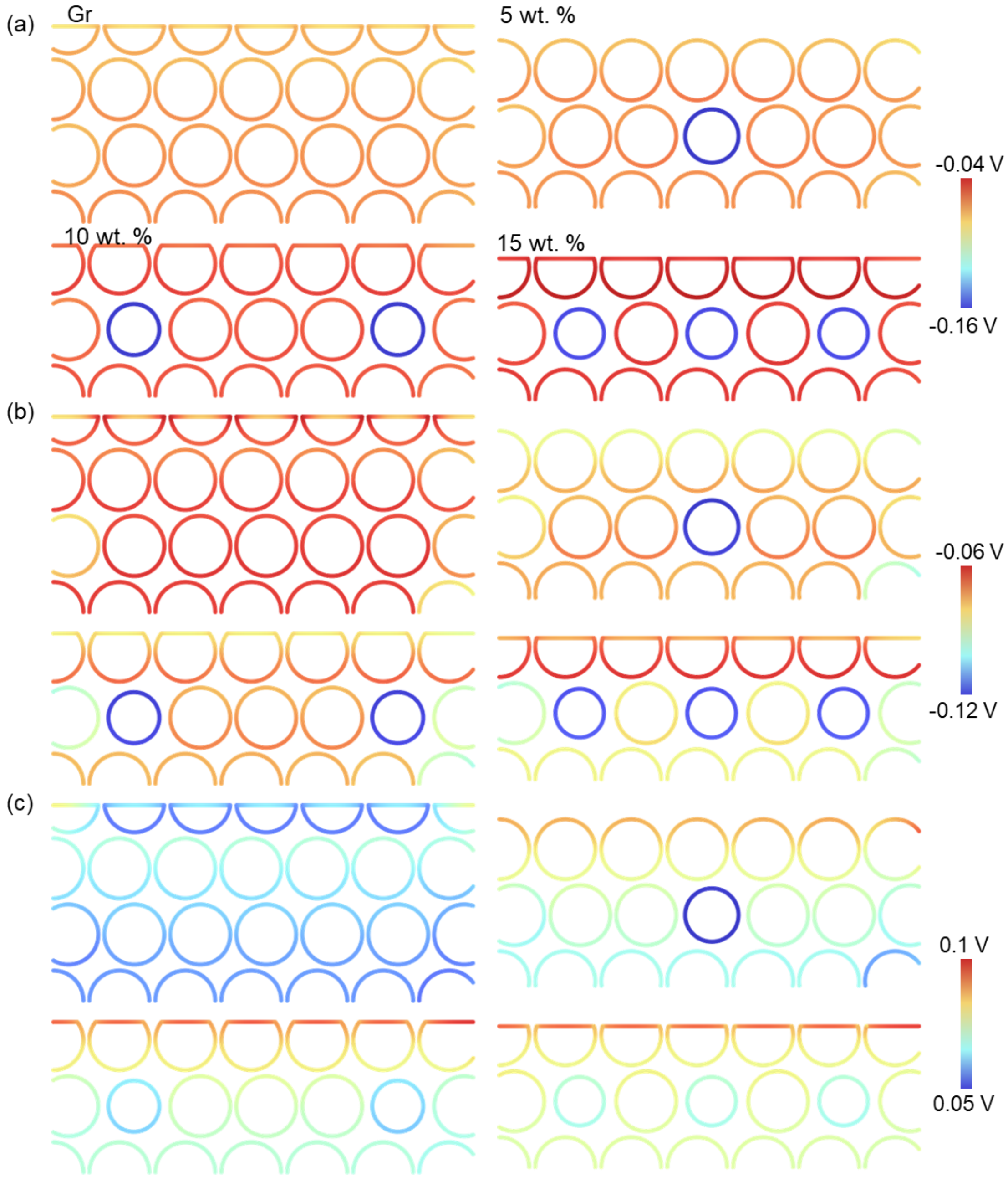

Figure S10. The detailed distribution of the differences between the overpotentials of the SiO/Gr composite anodes with various SiO wt. %s (0 wt.%, 5 wt. %, 10 wt. %, and 15 wt. %) during the charging/discharging cycling process at time points (a) t_1, (b) t_2, and (c) t_3 (defined in Figure S8). Note: the anode theoretical maximum capacities in these cases are the same.

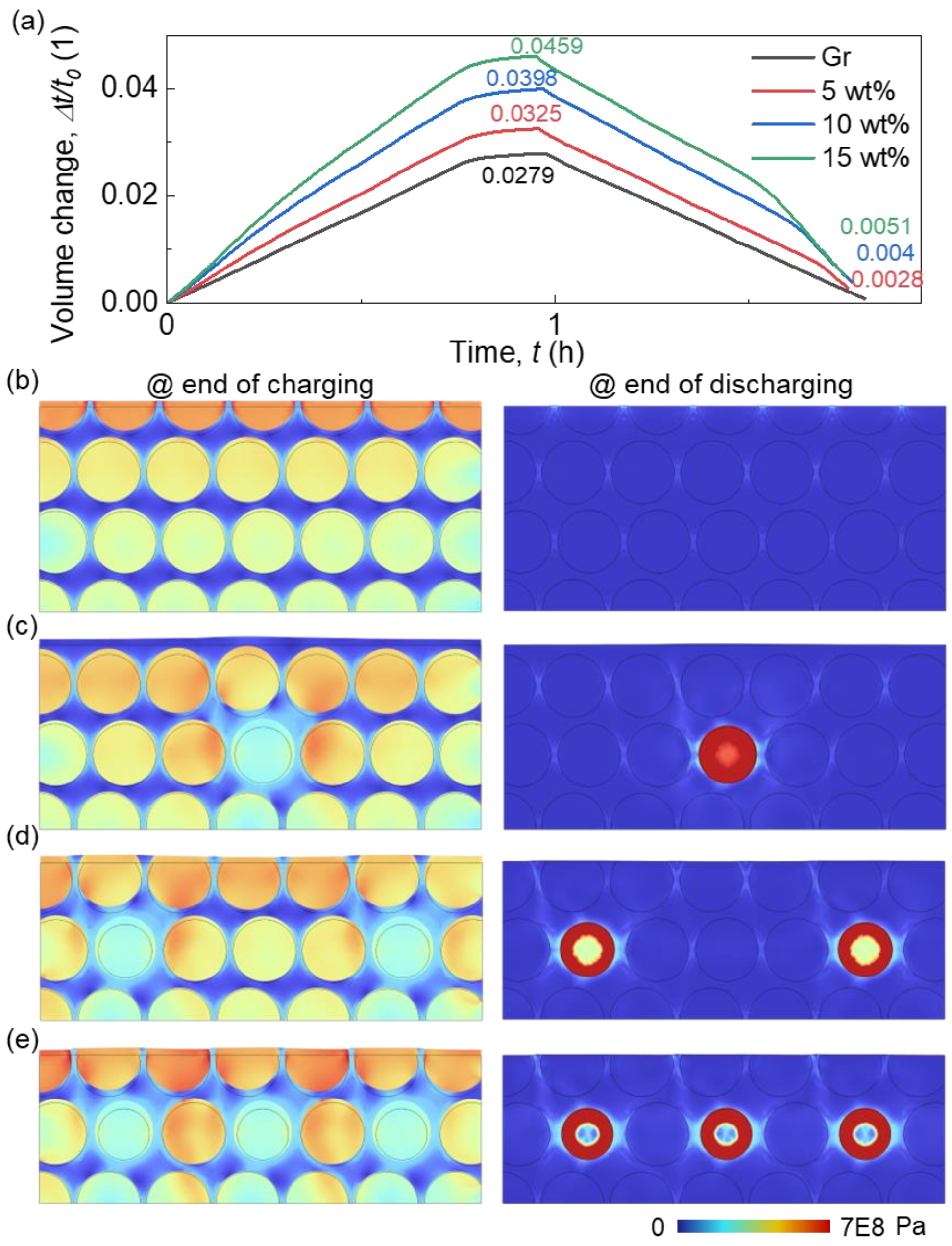

Figure S11. Computation results of the SiO/Gr composite anodes with various SiO wt. %s during the charging/discharging cycling process about (a) the overall volume changes with inset numbers indicating the ultimate values and residual values; the detailed Von Mises stress distributions at the end of charging and discharging processes of the cells with (b) 0 wt.%, (c) 5 wt. %, (d) 10 wt. %, and (e) 15 wt. %. Note: the anode theoretical maximum capacities in these cases are the same.

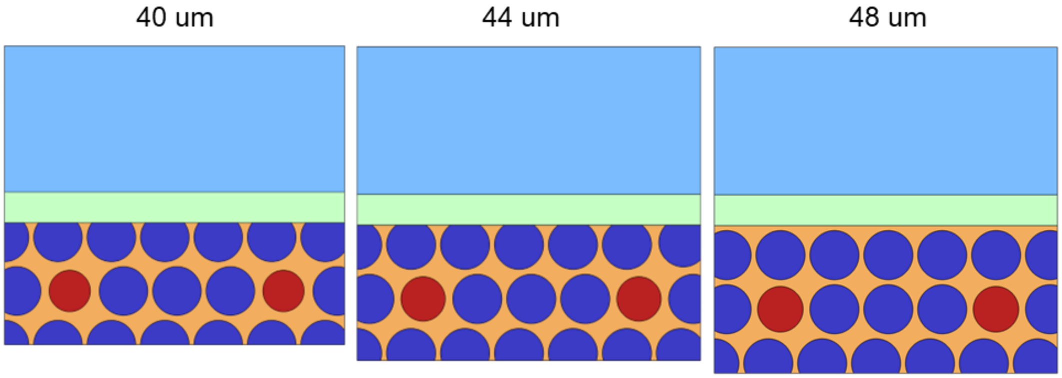

Figure S12. RVE models with same SiOw t. % (10 wt. %) but different thicknesses of anode.

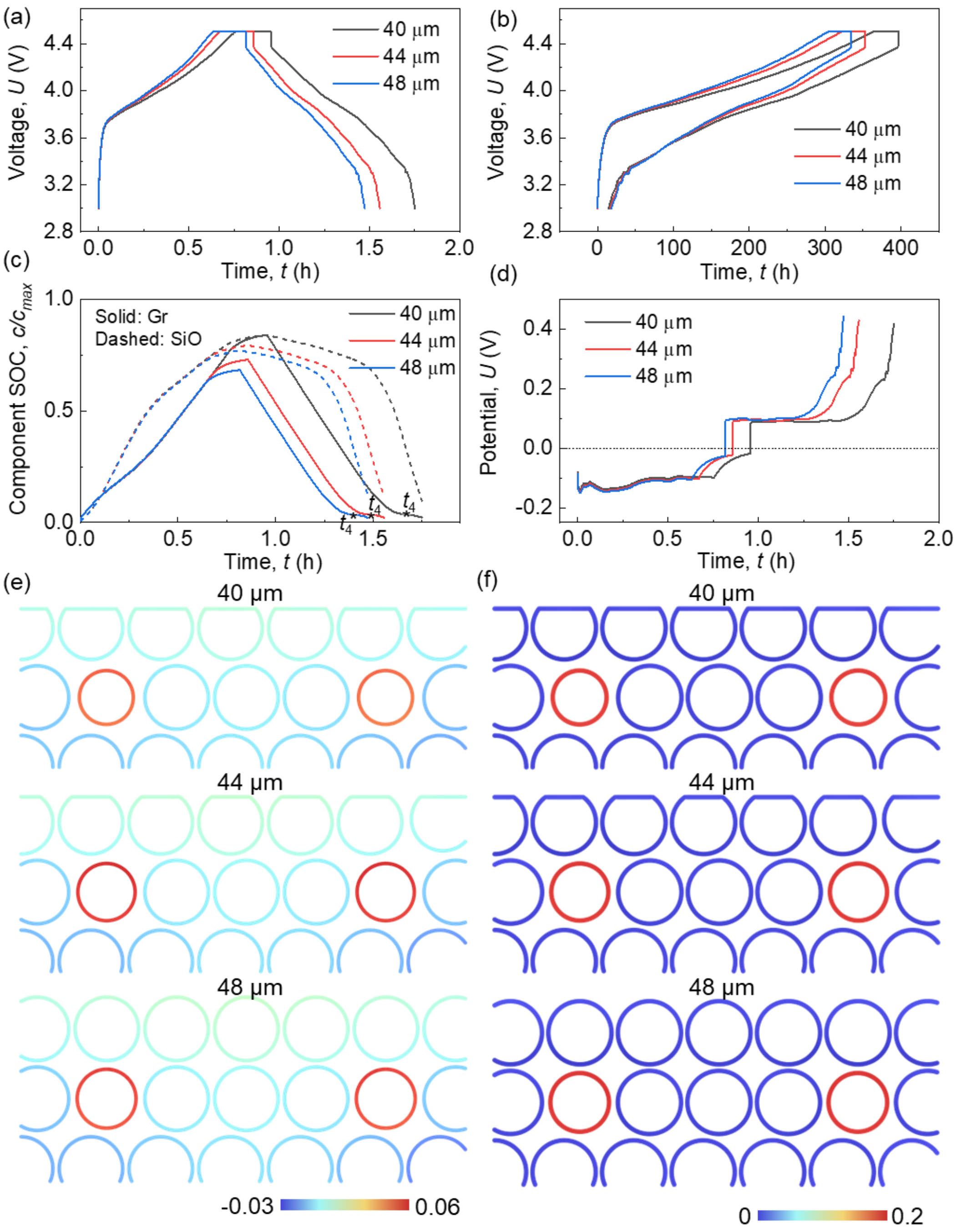

Figure S13. Computation results of the SiO/Gr composite anodes with various anode thicknesses and same SiO wt. % (10 wt. %) during the charging/discharging cycling process about (a) voltage vs. time profiles; (b) voltage vs. capacity profiles; (c) component SOCs of SiO and Gr materials;

(d) total polarization profiles; detailed distribution of (e) the difference between surface potential and average potential, $E_{surf} - E_{ave}$, and (f) the overpotential, at time point t_4 (indicated in figure S13 (c)).

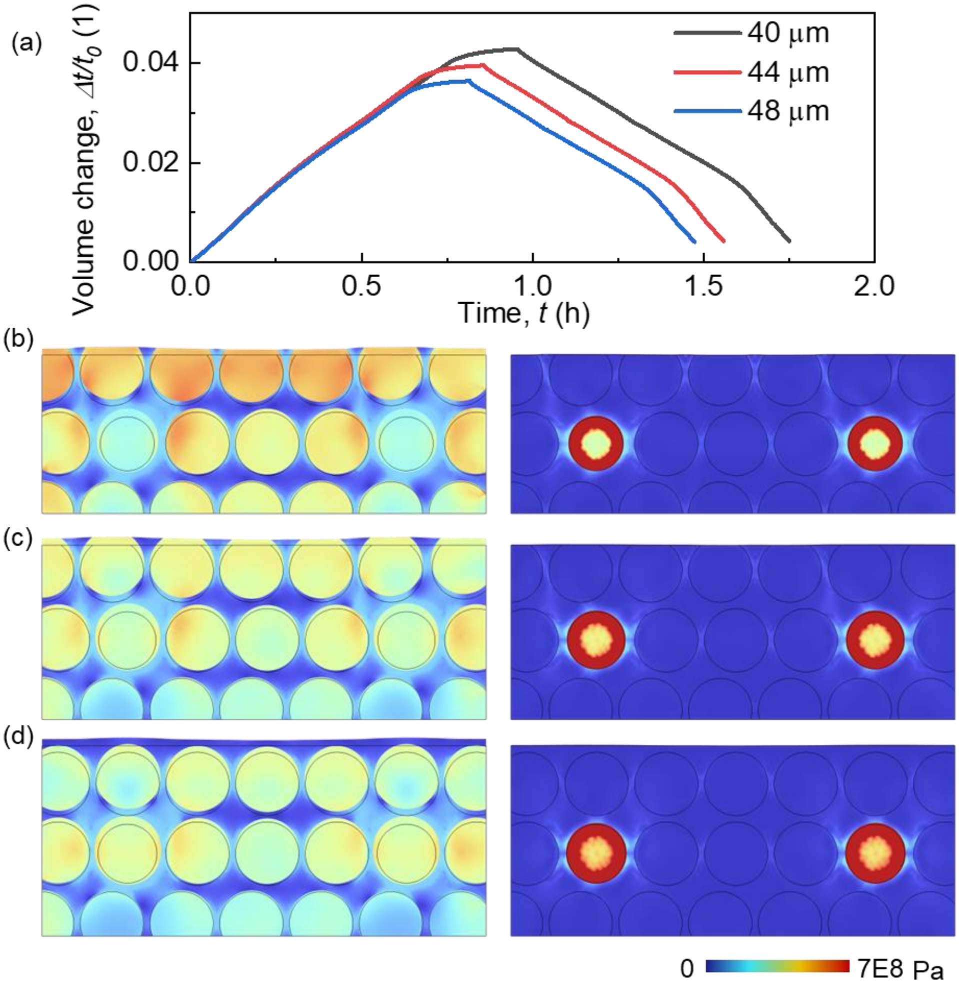

Figure S14. Computation results of the SiO/Gr composite anodes with various anode thicknesses and same SiO wt. % (10 wt. %) during the charging/discharging cycling process about (a) the overall volume changes; the detailed Von Mises stress distributions at the end of charging of the cells with anode thickness of (b) 40 μm, (c) 44 μm, and (d) 48 μm.

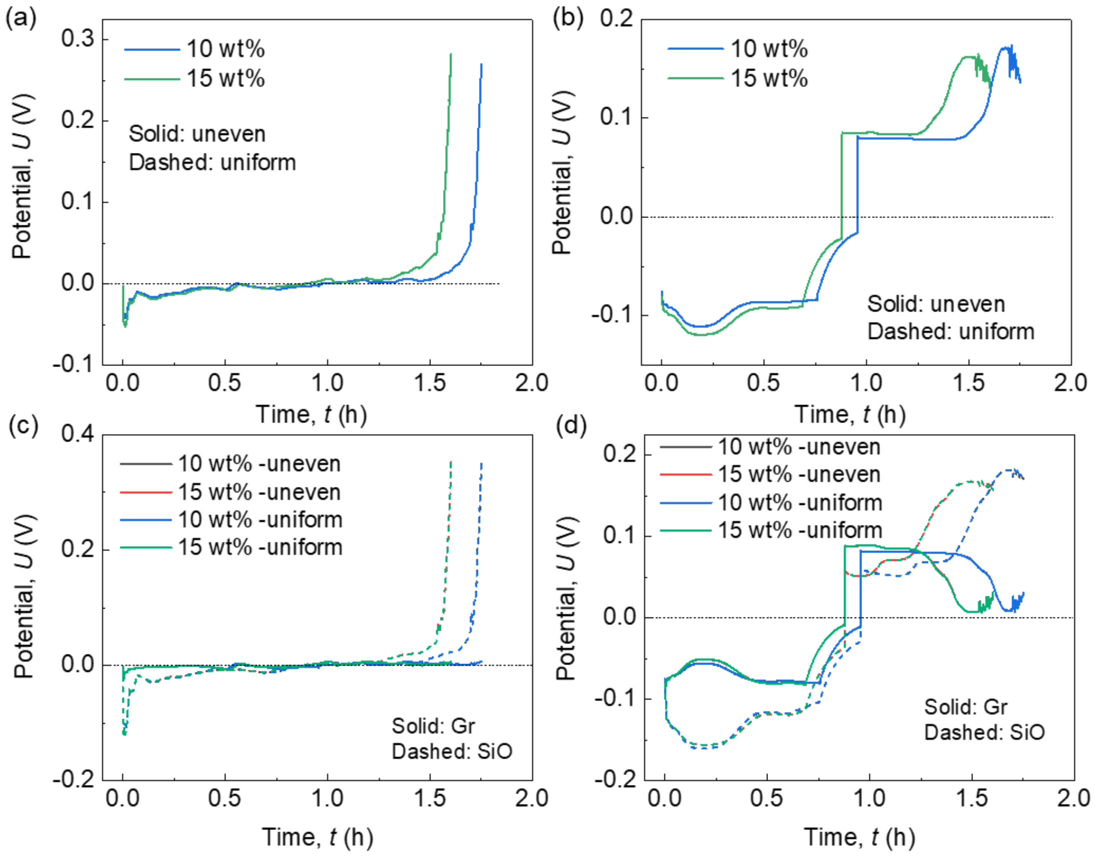

Figure S15. Computation results of the SiO/Gr composite anodes with two SiO wt. %s (10 wt. % and 15 wt. %) considering two types of SiO distribution unevenness during the charging/discharging cycling process about the polarization components of (a) diffusion polarization in solid phase and (b) activation overpotential. The polarization profiles in component materials (SiO and Gr) for (c) diffusion polarization in solid phase and (d) activation overpotential. Note: the anode thicknesses in these cases are the same.

.

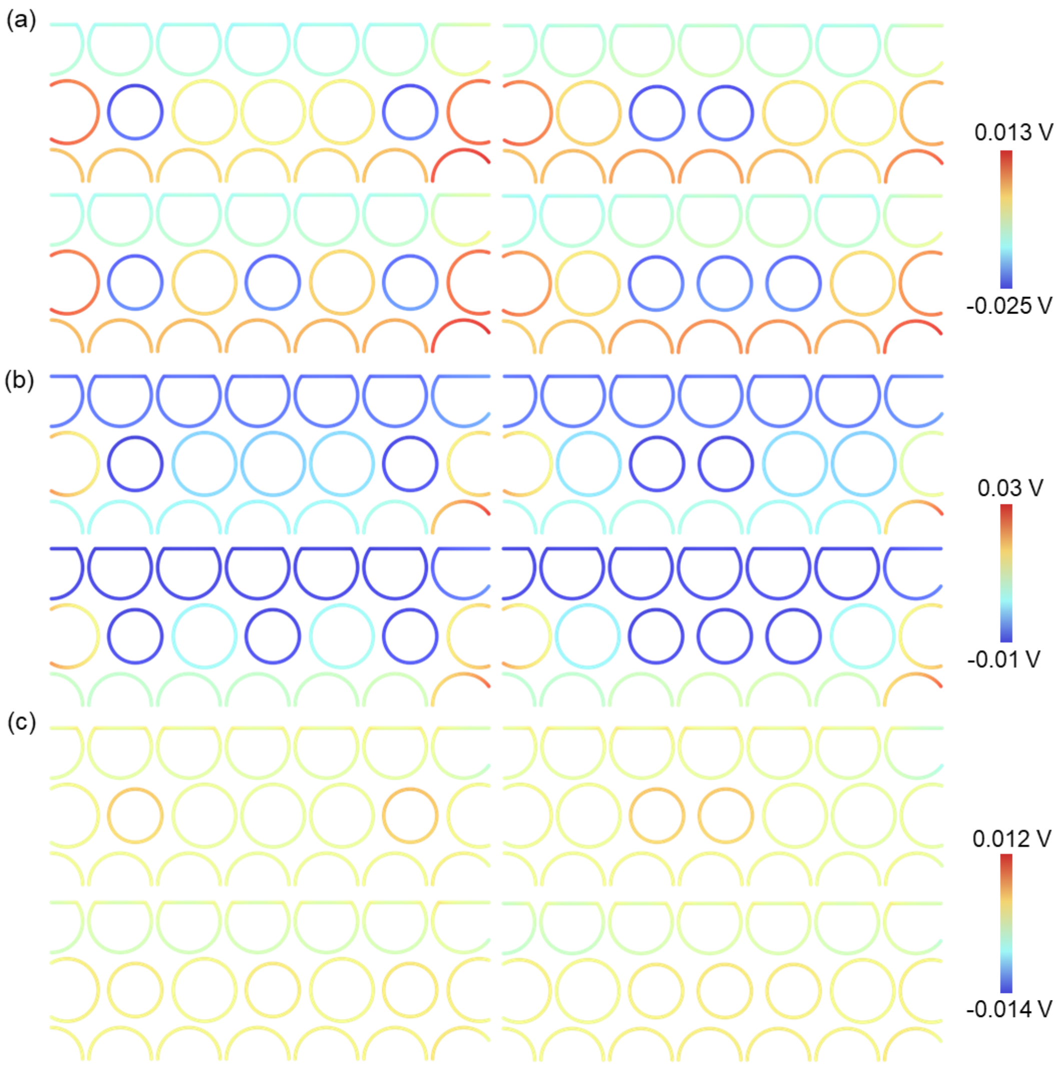

Figure S16. The detailed distribution of the differences between surface potential and average potential, E_{surf} - E_{ave}, of the SiO/Gr composite anodes with two SiO wt. %s (10 wt. % and 15 wt. %) considering two types of SiO distribution unevenness during the charging/discharging cycling process at time points (a) t_1, (b) t_2, and (c) t_3 (defined in Figure S1). Note: the anode thicknesses in these cases are the same.

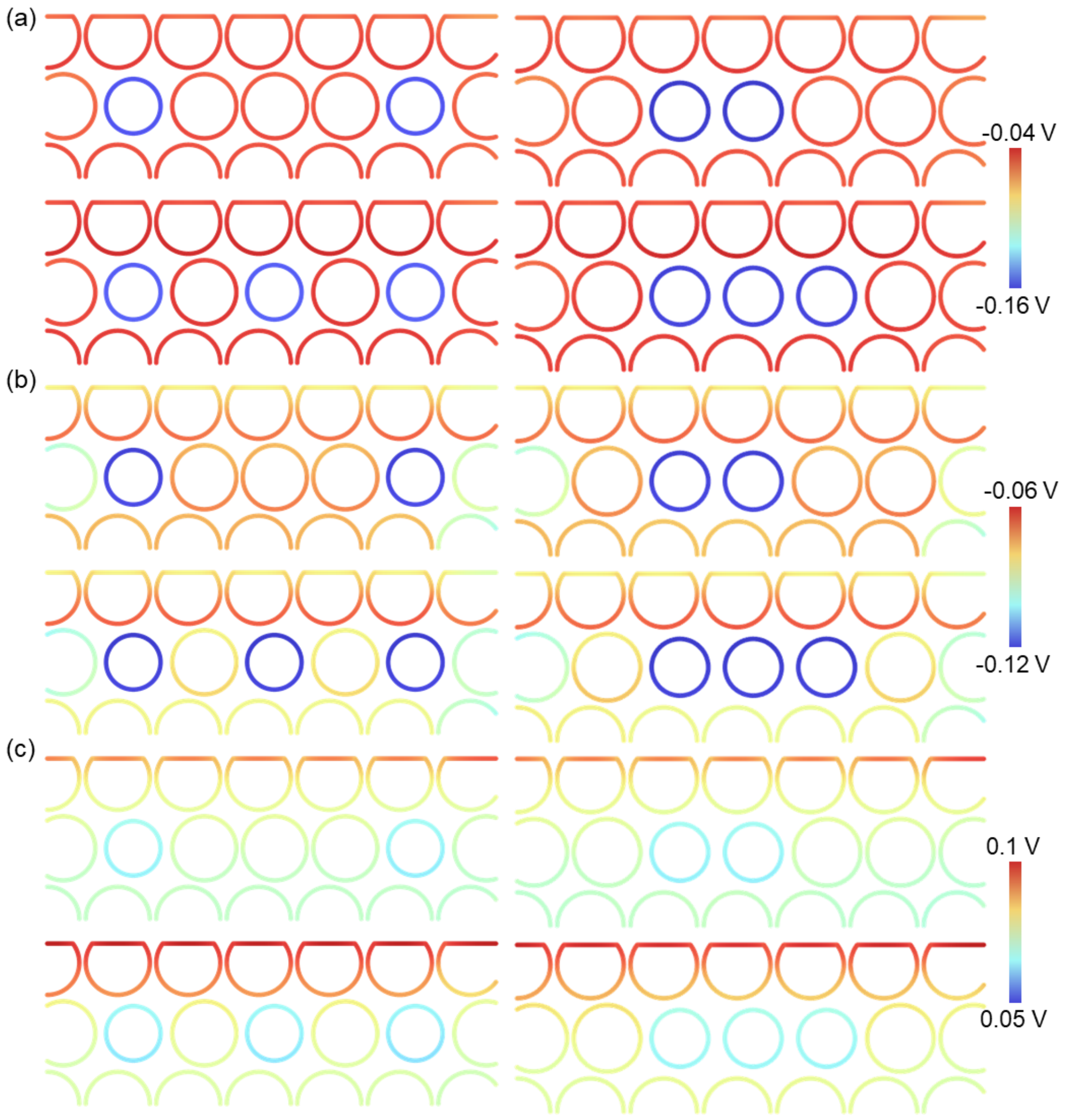

Figure S17. The detailed distribution of the differences between the overpotentials of the SiO/Gr composite anodes with two SiO wt. %s (10 wt. % and 15 wt. %) considering two types of SiO distribution unevenness during the charging/discharging cycling process at time points (a) t_1, (b) t_2, and (c) t_3 (defined in Figure S1). Note: the anode thicknesses in these cases are the same.

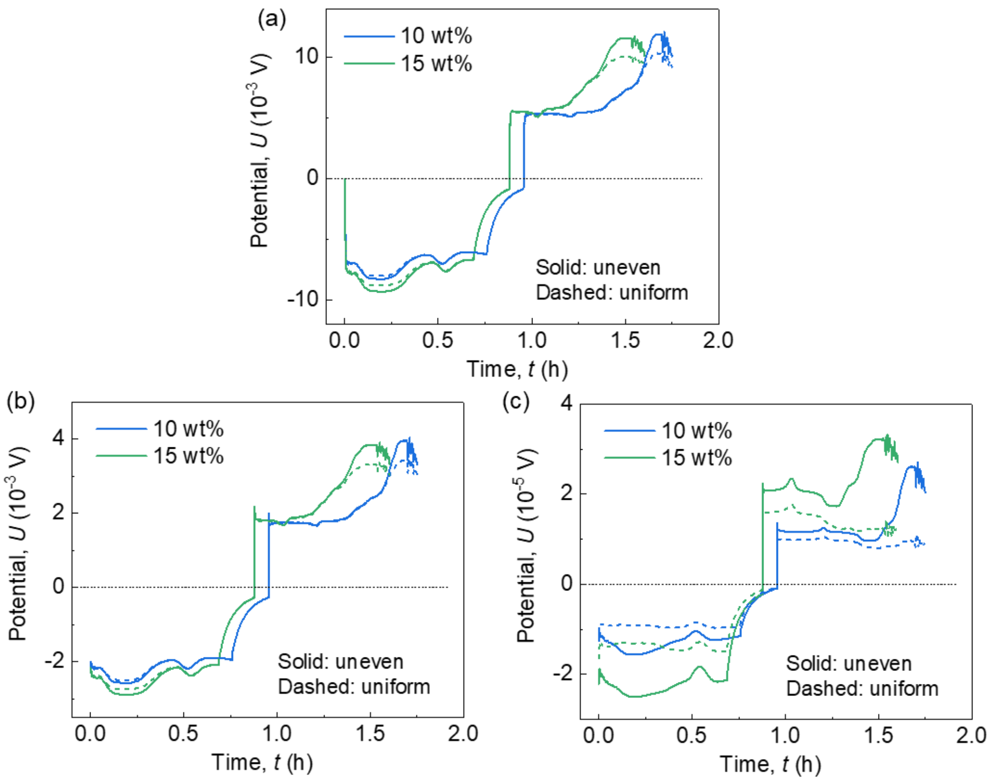

Figure S18. Computation results of the SiO/Gr composite anodes with two SiO wt. %s (10 wt. % and 15 wt. %) considering two types of SiO distribution unevenness during the charging/discharging cycling process about the polarization components of (a) diffusion polarization in liquid phase and the ohmic potential drops in (b) liquid phase and (c) solid phase. Note: the anode thicknesses in these cases are the same.

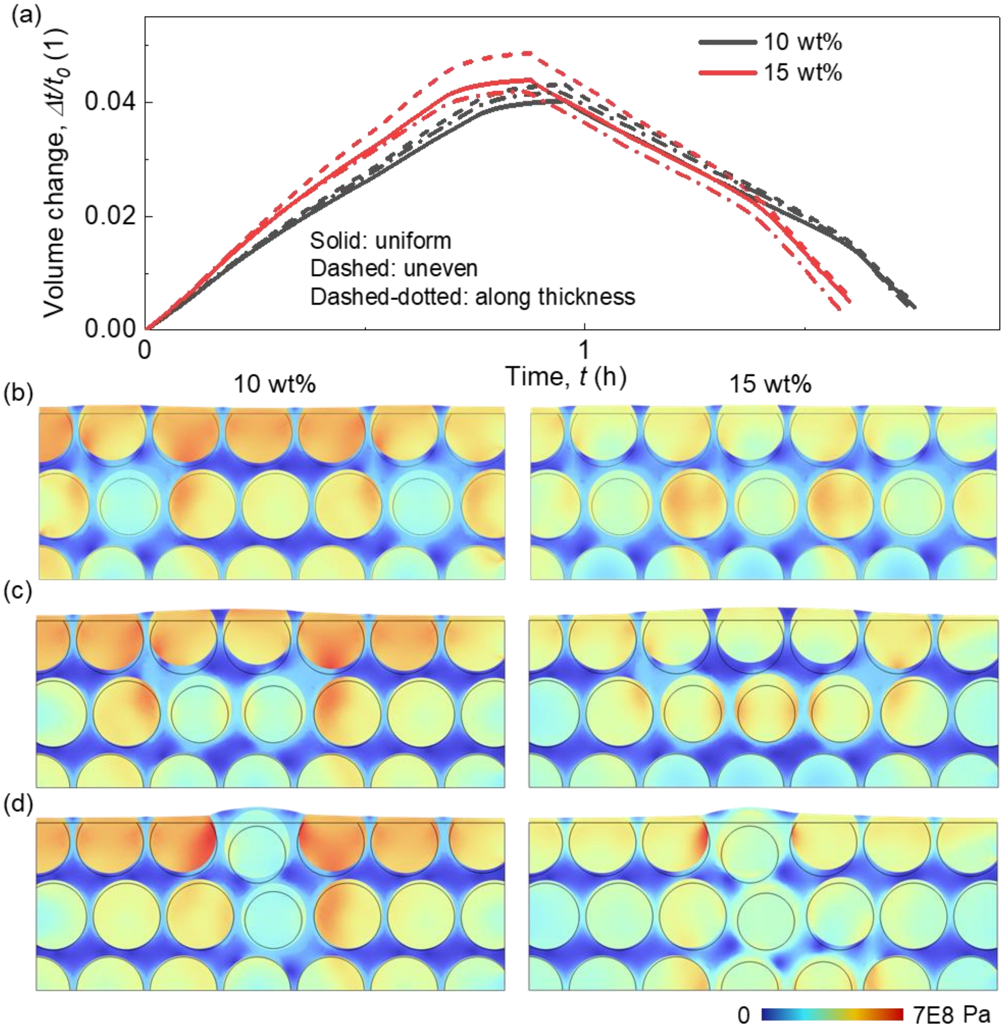

Figure S19. Computation results of the SiO/Gr composite anodes with two SiO wt. %s (10 wt. % and 15 wt. %) during the charging/discharging cycling process considering SiO distribution unevenness and directions about (a) the overall volume changes; the detailed Von Mises stress distributions at the end of charging of the cells with SiO particles (b) evenly distributed along the in-plane direction, (c) unevenly distributed along the in-plane direction, and (d) distributed along the thickness direction. Note: the anode theoretical maximum capacities in these cases are the same.

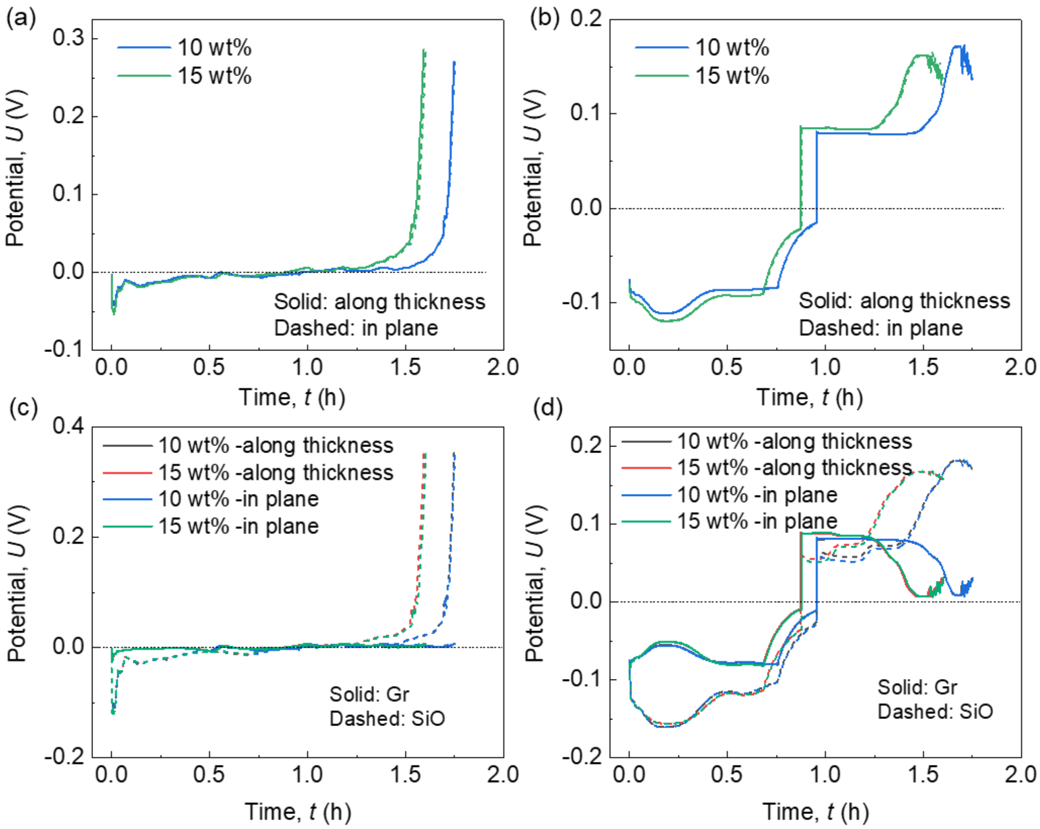

Figure S20. Computation results of the SiO/Gr composite anodes with two SiO wt. %s (10 wt. % and 15 wt. %) considering two types of SiO distribution directions during the charging/discharging cycling process about the polarization components of (a) diffusion polarization in solid phase and (b) activation overpotential. The polarization profiles in component materials (SiO and Gr) for (c) diffusion polarization in solid phase and (d) activation overpotential. Note: the anode thicknesses in these cases are the same.

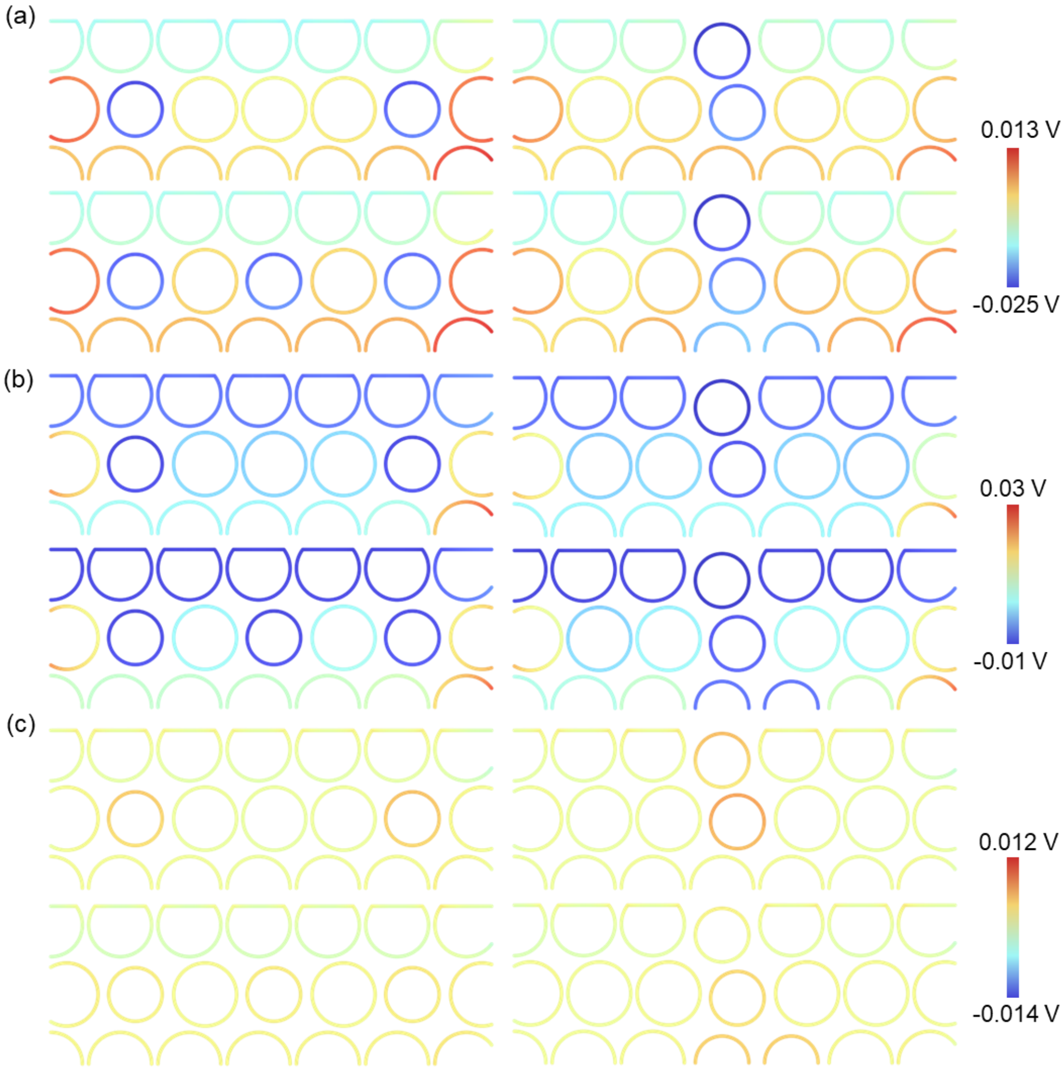

Figure S21. The detailed distribution of the differences between surface potential and average potential, $E_{surf} - E_{ave}$, of the SiO/Gr composite anodes with two SiO wt. %s (10 wt. % and 15 wt. %) considering two types of SiO distribution directions during the charging/discharging cycling process at time points (a) t_1, (b) t_2, and (c) t_3 (defined in Figure S1). Note: the anode thicknesses in these cases are the same.

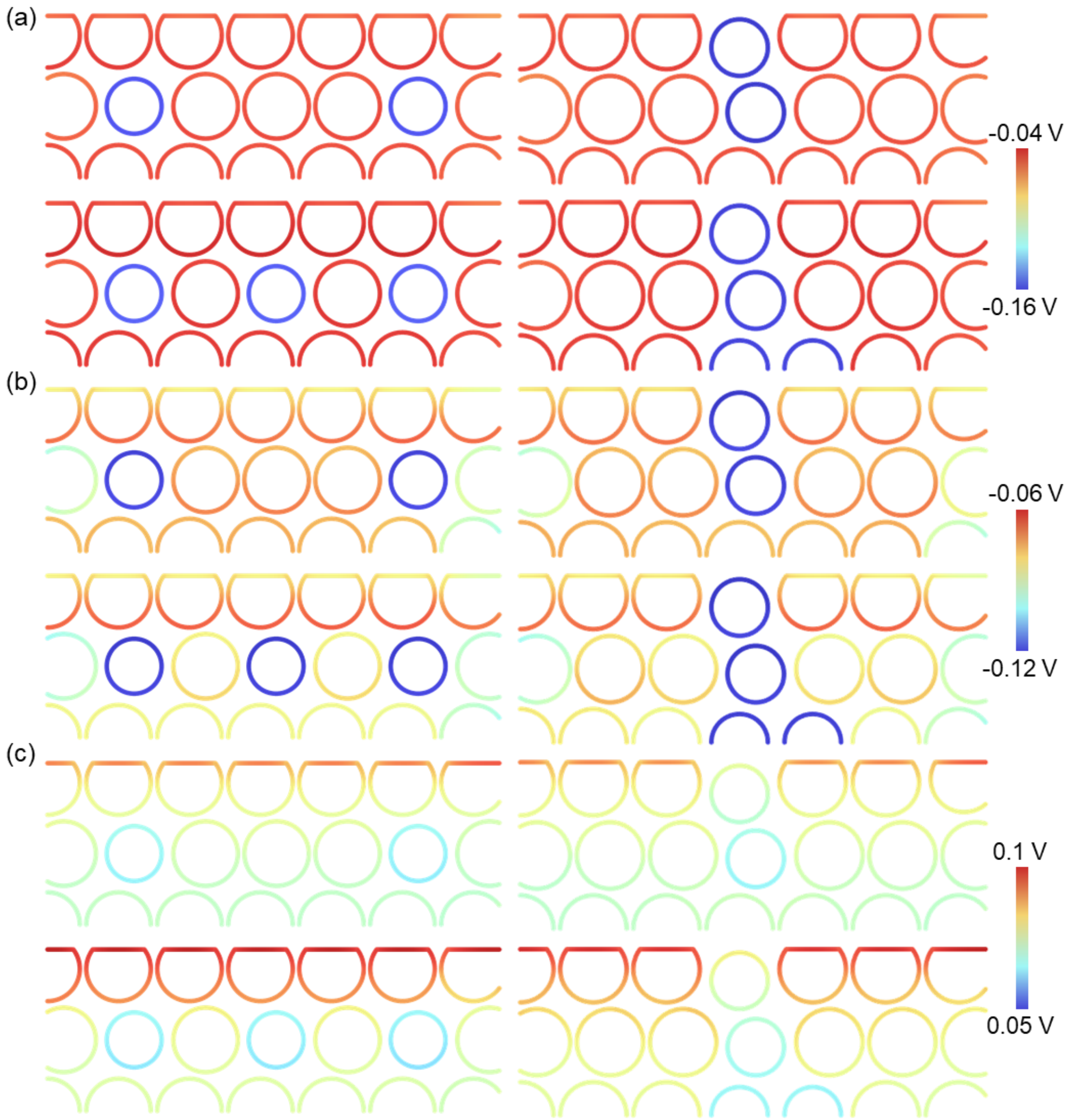

Figure S22. The detailed distribution of the differences between the overpotentials of the SiO/Gr composite anodes with two SiO wt. %s (10 wt. % and 15 wt. %) considering two types of SiO distribution directions during the charging/discharging cycling process at time points (a) t_1, (b) t_2, and (c) t_3 (defined in Figure S1). Note: the anode thicknesses in these cases are the same.

Table S1 The theoretical N/P ratio values for each parametric case

	N/P ratio values			
	Pure Gr	5 wt. % SiO	10 wt. % SiO	15 wt. % SiO
Same anode thickness	1.018	1.090	1.213	1.336
Same anode capacity	1.213	1.213	1.213	1.213
SiO uneven distribution	\	\	1.213	1.336
SiO thickness direction distribution	\	\	1.213	1.336
